# CircPDE5A-encoded novel regulator of the PI3K/AKT pathway inhibits esophageal squamous cell carcinoma progression by promoting USP14-mediated de-ubiquitination of PIK3IP1

**DOI:** 10.1186/s13046-024-03054-3

**Published:** 2024-04-24

**Authors:** Kai Lei, Ruihao Liang, Jialu Liang, Nan Lu, Jing Huang, Ke Xu, Binghua Tan, Kexi Wang, Yicheng Liang, Wenjian Wang, Huayue Lin, Minghui Wang

**Affiliations:** 1 Guangdong Provincial Key Laboratory of Malignant Tumor Epigenetics and Gene Regulation, Sun Yat-Sen Memorial Hospital, Sun Yat-Sen University, Guangzhou, 510120 China; 2 Department of Thoracic Surgery, Sun Yat-Sen Memorial Hospital, Sun Yat-Sen University, Guangzhou, 510120 China; 3 Nanhai Translational Innovation Center of Precision Immunology, Sun Yat-Sen Memorial Hospital, Foshan, 528200 China; 4 Breast Tumor Center, Sun Yat-Sen Memorial Hospital, Sun Yat-Sen University, Guangzhou, 510120 China

**Keywords:** Esophageal squamous cell carcinoma, PI3K/AKT pathway, Circular RNA, CircPDE5A, Nanoparticles

## Abstract

**Background:**

Esophageal squamous cell carcinoma (ESCC) is a common gastrointestinal tumor and has become an important global health problem. The PI3K/AKT signaling pathway plays a key role in the development of ESCC. CircRNAs have been reported to be involved in the regulation of the PI3K/AKT pathway, but the underlying mechanisms are unclear. Therefore, this study aimed to identify protein-coding circRNAs and investigate their functions in ESCC.

**Methods:**

Differential expression of circRNAs between ESCC tissues and adjacent normal tissues was identified using circRNA microarray analysis. Thereafter, LC–MS/MS was used to identify circPDE5A-encoded novel protein PDE5A-500aa. Molecular biological methods were used to explore the biological functions and regulatory mechanisms of circPDE5A and PDE5A-500aa in ESCC. Lastly, circRNA-loaded nanoplatforms were constructed to investigate the therapeutic translation value of circPDE5A.

**Results:**

We found that circPDE5A expression was down-regulated in ESCC cells and tissues and that it was negatively associated with advanced clinicopathological stages and poorer prognosis in ESCC. Functionally, circPDE5A inhibited ESCC proliferation and metastasis in vitro and in vivo by encoding PDE5A-500aa, a key regulator of the PI3K/AKT signaling pathway in ESCC. Mechanistically, PDE5A-500aa interacted with PIK3IP1 and promoted USP14-mediated de-ubiquitination of the k48-linked polyubiquitin chain at its K198 residue, thereby attenuating the PI3K/AKT pathway in ESCC. In addition, Meo-PEG-*S–S*-PLGA-based reduction-responsive nanoplatforms loaded with circPDE5A and PDE5A-500aa plasmids were found to successfully inhibit the growth and metastasis of ESCC in vitro and in vivo.

**Conclusion:**

The novel protein PDE5A-500aa encoded by circPDE5A can act as an inhibitor of the PI3K/AKT signaling pathway to inhibit the progression of ESCC by promoting USP14-mediated de-ubiquitination of PIK3IP1 and may serve as a potential target for the development of therapeutic agents.

**Graphical Abstract:**

The novel protein PDE5A-500aa encoded by circPDE5A can act as an inhibitor of the PI3K/AKT signaling pathway to inhibit the progression of esophageal squamous cell carcinoma.

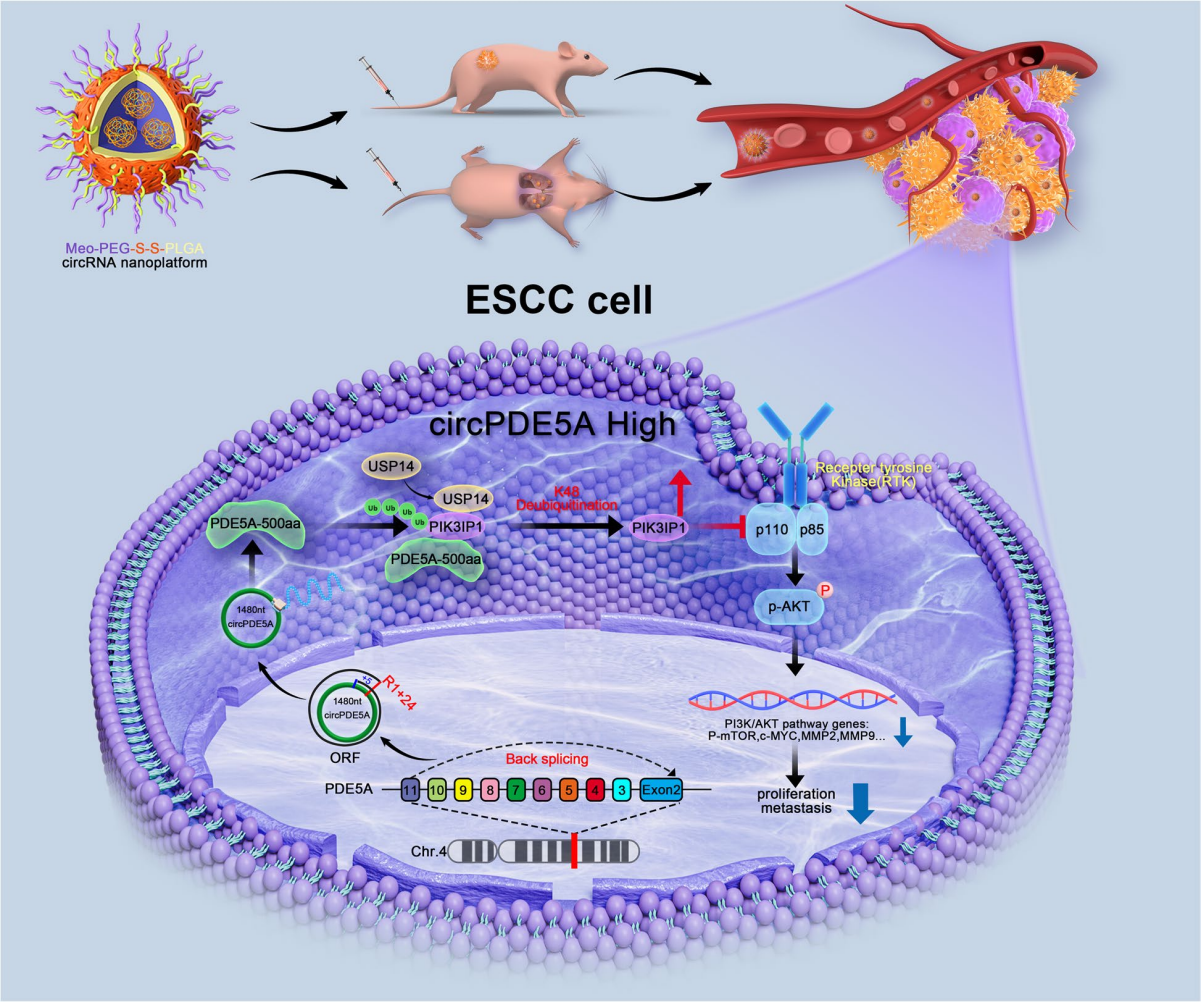

**Supplementary Information:**

The online version contains supplementary material available at 10.1186/s13046-024-03054-3.

## Introduction

Esophageal cancer (EC) is the eighth most common malignancy and the sixth most common cause of cancer-related deaths worldwide [1]. Esophageal squamous cell carcinoma (ESCC) is the most common pathological type of esophageal cancer, accounting for more than 90% [2]. ESCC is characterized by rapid tumor growth and high metastasis and recurrence rates, resulting in poor prognosis of ESCC patients. With the development of several treatment modalities, such as minimally invasive surgery, targeted therapy, and immunotherapy, the 5-year survival rate for ESCC patients has improved but remains suboptimal [3, 4]. Therefore, there is an urgent need to elucidate the comprehensive molecular mechanisms involved in the occurrence and development of ESCC and to identify novel therapeutic targets to further improve the prognosis of ESCC patients.

Circular RNAs (circRNAs) are specialized multifunctional non-coding RNAs that are cyclized by covalent reverse splicing of pre-mRNAs [5]. CircRNAs are characterized by high stability and conservatism and can serve as important regulators in many physiological and pathological processes [6]. Most studies on circRNAs have focused on the role of dysregulated circRNAs as microRNA (miRNA) sponges or regulators of classical signaling pathways involved in tumor progression [7, 8]. Recently, increasing attention has been drawn towards a previously unknown protein-coding ability of specific circRNAs with N6-methyladenosine (m6A), internal ribosomal entry sites (IRES), and open reading frames (ORFs) [9]. Huang et al. [10] showed that the cryptic peptides translated by circFAM53B efficiently primed naive CD4 + and CD8 + T cells in an antigen-specific manner and induced anti-tumor immunity. In a previous study, we reported that the circUBE4E-173aa novel protein encoded by circUBE4B interacts with MAPK1 to activate the MAPK/ERK signaling pathway to promote ESCC progression [11]. However, the role of protein-coding circRNAs in ESCC progression remains largely unknown.

The phosphatidylinositol 3-kinase (PI3K)/AKT signaling pathway is involved in a variety of physiological and pathological processes, including tumor progression [12, 13]. It plays an important role in regulating the proliferation, survival, metastasis, and metabolism of ESCC cells [14–16] and is associated with the poor prognosis of ESCC patients [17–19]. In addition, the circRNA/PI3K/AKT axis is an important regulatory pathway for tumor development [20]. For example, circRNA_0000392 promotes colorectal cancer progression through the miR-193a-5p/PIK3R3/AKT axis [21], and circNRIP1 acts as a miRNA-149-5p sponge to promote gastric cancer progression through the AKT1/mTOR pathway [22]. However, information on the circRNAs involved in the regulation of the PI3K/AKT signaling pathway in ESCC remains limited.

In this study, PDE5A-500aa encoded by circPDE5A was identified as a negative regulator of the PI3K/AKT pathway, which inhibits the progression of ESCC by modulating the post-translational modification of PIK3IP1. What's more, we have successfully constructed nanoplatforms loaded with circPDE5A and PDE5A-500aa, which can effectively inhibit the proliferation and metastasis of ESCC. These results suggest that dysregulated expressed circRNAs can affect tumor biological processes by translating proteins. They also uncover the potential of circPDE5A/PDE5A-500aa as promising therapeutic targets for ESCC.

## Materials and methods

### Cell lines and cell culture

HEK293T cells, human normal esophageal epithelial cells (HEEC), and human ESCC cells (TE1, ECA109, KYSE30, KYSE150, KYSE180, KYSE450, and EC18) were sourced from the Cell Bank of the Chinese Academy of Sciences (Shanghai, China). All cells were maintained in DMEM medium (Gibco, Billings, MT, USA) supplemented with 10% fetal bovine serum (Gibco) and 1% penicillin–streptomycin (Biosharp, Hefei, China). The cells were cultured in a humidified incubator at 37 °C with 5% CO_2_. Authentication of all cell lines was performed through a short tandem repeat DNA profiling test, and they tested negative for mycoplasma contamination.

### Human samples

Tumor tissues and adjacent normal tissues were gathered from a cohort of 100 patients with ESCC who underwent surgical treatment at the Department of Thoracic Surgery, Sun Yat-Sen Memorial Hospital, Sun Yat-Sen University, spanning the years 2013 to 2019. These specimens were utilized in subsequent experiments such as reverse transcription-quantitative polymerase chain reaction (RT-qPCR), fluorescence in situ hybridization (FISH), immunohistochemistry (IHC), and western blot (WB). Additionally, baseline clinical information for each patient was collected, and prognostic information was obtained through telephone interviews or outpatient follow-up. The inclusion criteria for this study encompassed: (1) patients confirmed with ESCC by postoperative histopathological examination; (2) patients who had not undergone chemotherapy, radiotherapy, targeted chemotherapy, or immunotherapy prior to surgery; (3) patients lacking distant metastases and a history of other tumors. Conversely, the exclusion criteria included: (1) patients diagnosed with esophageal adenocarcinoma or benign esophageal lesions; (2) patients with incomplete baseline clinical data or postoperative follow-up information. Approval for the study was granted by the Medical Ethics Committee (SYSKY-2023–1143-01) of Sun Yat-Sen Memorial Hospital, Sun Yat-Sen University.

### Animals

All in vivo studies adhered to protocols approved by the Institutional Animal Care and Use Committee of Sun Yat-sen University (SYSU-IACUC-2023–001965). BALB/c nude mice, aged 4–6 weeks, were procured from the Animal Research Centre of Sun Yat-sen University and were housed in accordance with institutional guidelines.

### Preparation and characterizations of circRNA-loaded NPs

NPs loaded with circRNA were prepared by nanoprecipitation. Meo-PEG_5k_*-S–S-*PLGA_10k_ was procured from Xi'an Ruixi Biological Technology (China). Cationic lipid-like compound alkylmodified polyamidoamine (PAMAM) dendrimer (G0-C14) was synthesized through ring opening of 1,2-epoxytetradecane by generation 0 of (PAMAM) dendrimer according to our previous study [23].

In brief, 30 mg Meo-PEG_5k_*-S–S-*PLGA_10k_ was dissolved in 1 mL dimethylformamide (DMF). Simultaneously, circPDE5A plasmid (10 μg, 1 mg/mL deionized water solution) and G0-C14 (50 μL, 5 mg/mL DMF solution) were prepared and combined with the Meo-PEG_5k_*-S–S-*PLGA_10k_ solution. This mixture was then added dropwise to RNase-free water (5 mL) under continuous stirring (1,000 rpm). The resulting NP suspension was transferred to an ultrafiltration unit (MWCO 100 K; EMP Millipore, Billerica, MA, USA) for centrifugal purification to eliminate organic solvents and free compounds. After three washes with deionized water, the circRNA-loaded NPs were collected and suspended in PBS at a circRNA concentration of 10 μg/mL. The size and zeta potential of the NPs were assessed using dynamic light scattering (DLS; Malvern, Worcestershire, UK), and the NP morphology was observed by transmission electron microscopy (TEM) (Tecnai G2 Spirit BioTWIN; FEI, Hillsboro, Oregon, USA). To determine the encapsulation efficiency (EE%) of circRNA, NPs loaded with Cy5-circRNA plasmid were prepared. The NP structure was disrupted by mixing the NP suspension with dimethyl sulfoxide (DMSO) at a volume ratio of 1/20, and the fluorescence intensity of NP was measured using a Synergy HT multimode microplate reader (BioTek, Winooski, VT, USA) to measure the fluorescence intensity of Cy5-circRNA plasmid encapsulated in NP. The EE% of Cy5-circRNA plasmid was calculated from the standard curve. The circRNA plasmid-loaded NPs, with a circRNA plasmid encapsulation efficiency of ∼80% and an average size of ∼100 nm, were employed for subsequent experiments by adjusting the loading amount.

### Formation of subcutaneous xenograft tumors and lung metastases

To initiate subcutaneous xenograft tumor formation, 4 × 10^6^ KYSE30 or ECA109 cells transfected with various vectors (vector, circPDE5A, sh-NC, sh-circPDE5A, circPDE5A-Mut, and PDE5A-500aa) were subcutaneously injected into BALB/c nude mice aged 4–6 weeks (*n* = 6). Tumor volume was calculated using the formula (V = 1/2 × L × W^2^, where L and W represent the long and short diameters of the tumor) and measured weekly. All mice were euthanized after 6 weeks, and tumors were excised and weighed. For the formation of lung metastases, 3 × 10^6^ KYSE30 cells transfected with different vectors (vector, circPDE5A, sh-NC, sh-circPDE5A, circPDE5A-Mut, and PDE5A-500aa) were injected into the tail vein of BALB/c nude mice aged 4–6 weeks (*n* = 6). At 6 weeks, photographs were captured using a Tanon ABL X6 Small Animal Live Imaging System (Tanon, Shanghai, China). Lung tissues were collected for hematoxylin and eosin (HE) staining after euthanasia, and the number of lung metastatic nodules was quantified in each sample.

### Cellular uptake

Confocal dishes were seeded with 3 × 10^4^ KYSE30 cells. Following a 24-h incubation period, the KYSE30 cells were subjected to treatments with PBS, naked Cy5-circPDE5A plasmid, and NPs loaded with Cy5-circPDE5A for 4 h. Subsequently, the KYSE30 cells from the three groups were fixed with 4% paraformaldehyde and examined using an Olympus FV3000 laser scanning confocal system (Olympus, Tokyo, Japan).

### Biodistribution

Naked Cy5-circPDE5A plasmid or NPs loaded with Cy5-circPDE5A were injected into subcutaneous xenograft tumor-bearing mice via the tail vein at a dose of 10 μg circPDE5A per mouse. After 24 h post-injection, whole-body images of the mice were acquired using a Tanon ABL X6 small animal live imaging system (Tanon). Following euthanasia, major organs and tumors were harvested and subjected to imaging.

### NPs loaded with circRNA inhibit ESCC proliferation and metastasis

For the construction of the subcutaneous xenograft tumor model, 4 × 10^6^ KYSE30 cells were subcutaneously injected into the lateral abdomen of mice. Upon reaching a tumor volume of 100–150 mm^3^, 48 mice were randomly divided into 6 groups for subsequent nanoparticle treatment experiments. To establish a lung metastasis model, 3 × 10^6^ KYSE30 cells were injected into the tail vein. After 3 weeks, 30 mice were randomly divided into 6 groups for the subsequent nanoparticle treatment experiments.

Mice-bearing subcutaneous and lung metastatic ESCC tumors were randomly allocated into 6 groups. They were injected via the tail vein with the following substances: PBS, vector-loaded NPs (10 μg), naked circPDE5A plasmid (10 μg), circPDE5A-loaded NPs (10 μg), circPDE5A-Mut-loaded NPs (10 μg), and PDE5A-500aa-loaded NPs (10 μg). The injections were given every 2 days, with two injections per cycle for a total of three consecutive cycles.

In the case of the subcutaneous tumor model, the mice's body weight and tumor volume were monitored every 4 days from the initiation of drug administration. Approximately one week after the completion of the treatment, the mice were euthanized and their tumors were extracted for both weighing and photography. The tumor tissues were fixed in 4% formaldehyde and utilized for IHC staining. For the lung metastasis model, mice were imaged using the Tanon ABL X6 Small Animal Live Imaging System (Tanon) approximately one week after the completion of treatment. Lung tissues were collected for IHC staining after euthanasia, and the number of pulmonary metastatic nodules was determined in each sample.

### Human circular RNA (circRNA) array analysis

Differential expression of circRNA was examined in three pairs of ESCC tissues and their corresponding adjacent normal tissues using the Arraystar Human circRNA Array analysis. All raw data generated from the microarray analysis have been deposited in the Gene Expression Omnibus (GEO) under accession number GSE250413. The total RNA from each sample was quantified using the NanoDrop ND-1000. Sample preparation and microarray hybridization were carried out following Arraystar's standard protocols.

In brief, total RNAs were subjected to digestion with Rnase R (Epicentre, Madison, WI, USA) to eliminate linear RNAs and enrich circRNAs. Subsequently, the enriched circRNAs underwent amplification and transcription into fluorescent cRNA using a random priming method (Arraystar Super RNA Labeling Kit; Arraystar, Rockville, MD, USA). The labeled cRNAs were then hybridized onto the Arraystar Human circRNA Array V2 (8 × 15 K; Arraystar). Following slide washing, the arrays were scanned using the Agilent Scanner G2505C (Santa Clara, CA, USA).

Agilent Feature Extraction v11.0.1.1 was employed for the analysis of acquired array images. Quantile normalization and subsequent data processing were conducted using the R software “limma” package. Differentially expressed circRNAs with statistical significance between the two groups were identified through Volcano Plot filtering. Additionally, Fold Change filtering was applied to pinpoint differentially expressed circRNAs between two samples. Hierarchical Clustering was performed to illustrate the distinguishable circRNA expression patterns among the samples.

### RNA sequencing

To conduct RNA sequencing, total RNA was isolated from ESCC cells overexpressing circPDE5A or control cells using the TRIzol Total RNA Isolation Kit (Takara, Shiga, Japan). The RNA samples underwent rigorous quality control, and ribosomal RNA was depleted to enrich for mRNA. Subsequently, the RNA was reverse-transcribed into complementary DNA (cDNA) using random primers. The resulting double-stranded cDNA was subjected to end repair, A-tailing, and ligation to sequencing connectors. cDNAs with a size range of 250 ~ 300 bp were selected using AMPure XP beads and then amplified by PCR. After purification of the PCR products with AMPure XP beads, the final library was obtained.

Following library construction, an initial quantification was carried out using a Qubit v2.0 Fluorometer, and the library was diluted to a concentration of 1.5 ng/μL. The insert size of the library was determined using an Agilent 2100 bioanalyzer, and experimental RT-qPCR was performed to accurately assess the effective concentration of the library, ensuring it exceeded 2 nM to maintain quality. Once the libraries passed the quality control, they were pooled based on effective concentration and the targeted downstream data volume for Illumina sequencing. Shanghai Origingene Bio-pharm Technology (China) performed the RNA sequencing and subsequent analysis. All raw data generated from the RNA sequencing have been deposited in the GEO database under the accession number GSE250323.

### Statistical analysis

Statistical analysis was conducted utilizing Statistical Product Service Solutions v26.0 (IBM Corporation, Armonk, NY, USA) and GraphPad Prism software v7.0 (GraphPad Software, La Jolla, CA, USA). Categorical variables were presented as numbers (percentages), and comparisons between two sets of data were performed using the chi-squared test (Fisher's exact test where applicable). Continuous variables were described by median (interquartile range), and comparisons between two groups were made using the *t*-test (Mann–Whitney *U* test where appropriate). Spearman correlation analysis was employed to examine the correlation between the two groups.

For survival analysis, the Kaplan–Meier (KM) method was applied, and assessments were carried out using the log-rank test. Overall survival (OS) and progression-free survival (PFS) were analyzed during the survival analysis.

## Results

### CircPDE5A is down-regulated in ESCC and is negatively associated with its poor prognosis

**Fig. 1:**
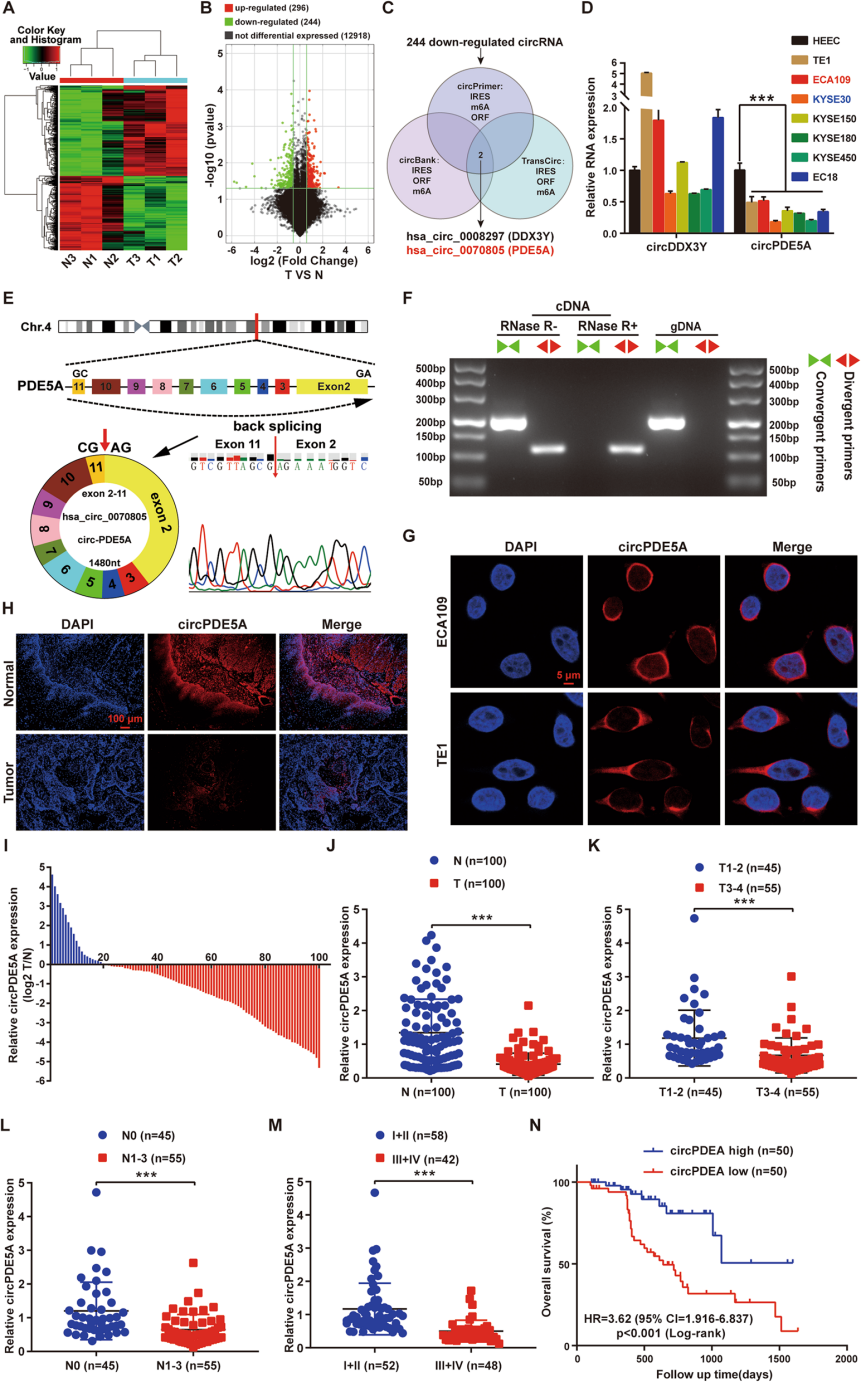
Characterization and clinical significance of circPDE5A. **A** Heatmap clustering displaying all dysregulated circRNAs. Rows represent circRNAs, and columns represent tissues. **B** Volcano plot comparing the fold change in circRNA level in three pairs of ESCC tissues versus adjacent normal tissues. Significantly up-regulated circRNAs are indicated by red dots, while significantly down-regulated circRNAs are indicated by green dots. **C** Venn diagram illustrating the selection of low-level circRNAs with protein-coding potential in ESCC. Selection criteria include the presence of internal ribosome entry site (IRES), open reading frame (ORF), and N6-methyladenosine (m6A) in predictions from circPrimer, circBank, and TransCirc databases. **D** RT-qPCR analysis of relative levels of circDDX3Y and circPDE5A in a normal esophageal epithelial cell line and various ESCC cell lines. **E** Genomic locations of PDE5A and circPDE5A. Sanger sequencing confirmed the reverse splice site of circPDE5A. **F** PCR and nucleic acid electrophoresis for determining the presence of circPDE5A using convergent and divergent primers in cDNA and gDNA, respectively. **G** RNA-FISH detection of subcellular localization of circPDE5A in ECA109 and TE1 cells. Bar represents 5 μm. **H** Representative images of RNA-FISH detection of circPDE5A level in ESCC tissues compared to adjacent normal tissues. Bar represents 100 μm. **I** Fold change of circPDE5A level in 100 pairs of ESCC tissues versus adjacent normal tissues. **J** RT-qPCR analysis to detect the relative levels of circPDE5A in 100 pairs of ESCC tissues versus adjacent normal tissues. **K–M** RT-qPCR analysis of the relative levels of circPDE5A in ESCC tissues with different T (**K**), N (**L**), and TNM (**M**) stages. **N** Kaplan–Meier analysis of the correlation between circPDE5A level and overall survival (OS) in patients with ESCC. ***P < 0.005

CircRNA microarray analysis of three pairs of ESCC tissues and adjacent normal tissues identified 296 up-regulated and 244 down-regulated circRNAs (Fig. 1A, B). ORF, IERS, and m6A are considered important structural features of protein-coding circRNAs [9]. Therefore, we used three databases to predict the translational potential of circRNAs and found that the down-regulated circDDX3Y (has_circ_0008297) and circPDE5A (has_circ_0070805) had ORFs, IERS, and m6A (Fig. 1C and Fig. S1). RT-qPCR showed that only circPDE5A was significantly down-regulated in the seven ESCC cell lines (Fig. 1D). Therefore, circPDE5A was further investigated in this study.

CircPDE5A is formed by reverse splicing exons 2–11 of the linear transcript of the phosphodiesterase 5A (PDE5A) gene, which is 1480 nt in length (Fig. 1E). PCR and nucleic acid electrophoresis showed that the linear PDE5A transcript of PDE5A could be amplified by convergent priming of complementary DNA (cDNA) and genomic DNA (gDNA), whereas the circular transcript of PED5A could only be amplified by convergent priming of cDNA (Fig. 1F). Additionally, Sanger sequencing further confirmed the presence of a reverse splice site of circPDE5A in the PCR products amplified by divergent primers (Fig. 1E). RNase R degradation assay showed that the linear PDE5A mRNA was susceptible, while the circPDE5A was resistant to RNase R treatment (Fig. S2A and B). In addition, actinomycin D assay showed that circPDE5A was more stable than the linear PDE5A mRNA (Fig. S2C). Additionally, nucleus/cytoplasmic RNA isolation (Fig. S2D and E) and FISH (Fig. 1G) assays showed that circPDE5A was enriched in the cytoplasm of ESCC cells. These results suggest that circPDE5A is a newly identified circRNA in ESCC.

FISH of tissue sections showed that circPDE5A expression was down-regulated in ESCC tissues (Fig. 1H). RT-qPCR analysis of ESCC tissues and adjacent normal tissues showed that circPDE5A expression was down-regulated in 80% of ESCC patients (Fig. 1I) and significantly lower in ESCC tissues than in adjacent normal tissues (Fig. 1 J). Clinical correlation analysis and Kaplan–Meier (KM) survival analysis showed that the low expression of circPDE5A was associated with the later clinicopathological stages (Fig. 1 K–M and Table 1) and poor prognosis (Fig. 1N and Fig. S2F) of ESCC patients. Furthermore, univariate and multivariate Cox regression analyses showed that low circPDE5A expression was an independent prognostic factor in ESCC patients (Table2). These results suggest that circPDE5A is lowly expressed in ESCC and is negatively associated with its poor prognosis.

**Table 1:** Relationship between general clinical characteristics and circPDE5A level in patients with ESCC (*n* = 100)

General clinical characteristics	CircPDE5A expression		*p*-value	statistics
	**High (*n* = 50)**	**Low (*n* = 50)**		
Gender (%)			1	0
Female	44 (88)	44 (88)		
Male	6 (12)	6 (12)		
Age (mean ± SD)	58.6 ± 10.367	60.22 ± 10.938	0.449	-0.760
BMI (mean ± SD)	23.103 ± 4.12	22.752 ± 3.62	0.652	0.453
Smoke history (%)	22 (44)	28 (52)	0.423	0.641
Alcohol history (%)	35 (70)	29 (58)	0.211	1.563
Histologic Grade (G stage) (%)			0.249	1.329
G1	9 (18)	5 (10)		
G2 + G3	41 (82)	45 (90)		
T stage			0***	17.818
T1 + T2	33 (66)	12 (24)		
T3 + T4	17 (34)	38 (76)		
N stage			0***	14.586
N0	32 (64)	13 (26)		
N1 + N2 + N3	18 (36)	37 (74)		
M stage			0.056	5.263^a^
M0	50 (100)	45 (90)		
M1	0 (0)	5 (10)		
TNM stage			0***	36.946
I + II	44 (88)	14 (28)		
III + IV	6 (12)	36 (72)		

*BMI* Body Mass Index, a Fisher exact probability test, *** *p* < 0.001

**Table 2:** Univariate and multivariate Cox-regression analysis of prognostic factors for patients with ESCC (*n* = 100)

Feature	n	Univariate analysis			Multivariate analysis		
		**HR**	**95% CI**	**p**	**HR**	**95% CI**	**p**
Gender							
Female	12	1					
Male	88	3.179	0.764–13.238	0.112			
Age							
< 65	72	1					
≥ 65	28	1.897	0.966–3.724	0.074			
BMI							
≥ 18.5	97	1					
< 18.5	3	2.881	1.196–6.939	0.036*	1.792	0.700–4.587	0.224
Smoking history							
No	52	1					
Yes	48	1.014	0.530–1.938	0.967			
Alcohol history							
No	36	1					
Yes	64	0.577	0.305–1.095	0.092			
Histologic grade							
G1	14	1					
G2 + G3	86	2.493	0.755–8.234	0.134			
T stage							
T1 + T2	45	1					
T3 + T4	55	1.386	0.723–2.657	0.326			
N stage							
N0	45	1					
N1 + N2 + N3	55	3.274	1.568–6.837	0.002**	2.527	0.829–7.346	0.069
M stage							
M0	95	1					
M1	5	3.155	1.105–9.012	0.032*	1.580	0.327–4.376	0.687
TNM stage							
I + II	58	1					
III + IV	42	2.853	1.462–5.565	0.002**	0.799	0.280–2.578	0.929
Expression of circPDE5A							
Low	50	1					
High	50	0.274	0.126–0.599	0.001**	0.354	0.139–0.875	0.034*
Expression of PDE5A-500aa							
Low	50	1					
High	50	0.203	0.079–0.522	0.001**	0.263	0.100–0.691	0.017*

n sample number, *HR* Hazard ratio, *CI* Confidence interval, *BMI* Body Mass Index * *p* < 0.05, ** *p* < 0.01

### CircPDE5A inhibits ESCC cell proliferation and metastasis in vitro and in vivo

**Fig. 2:**
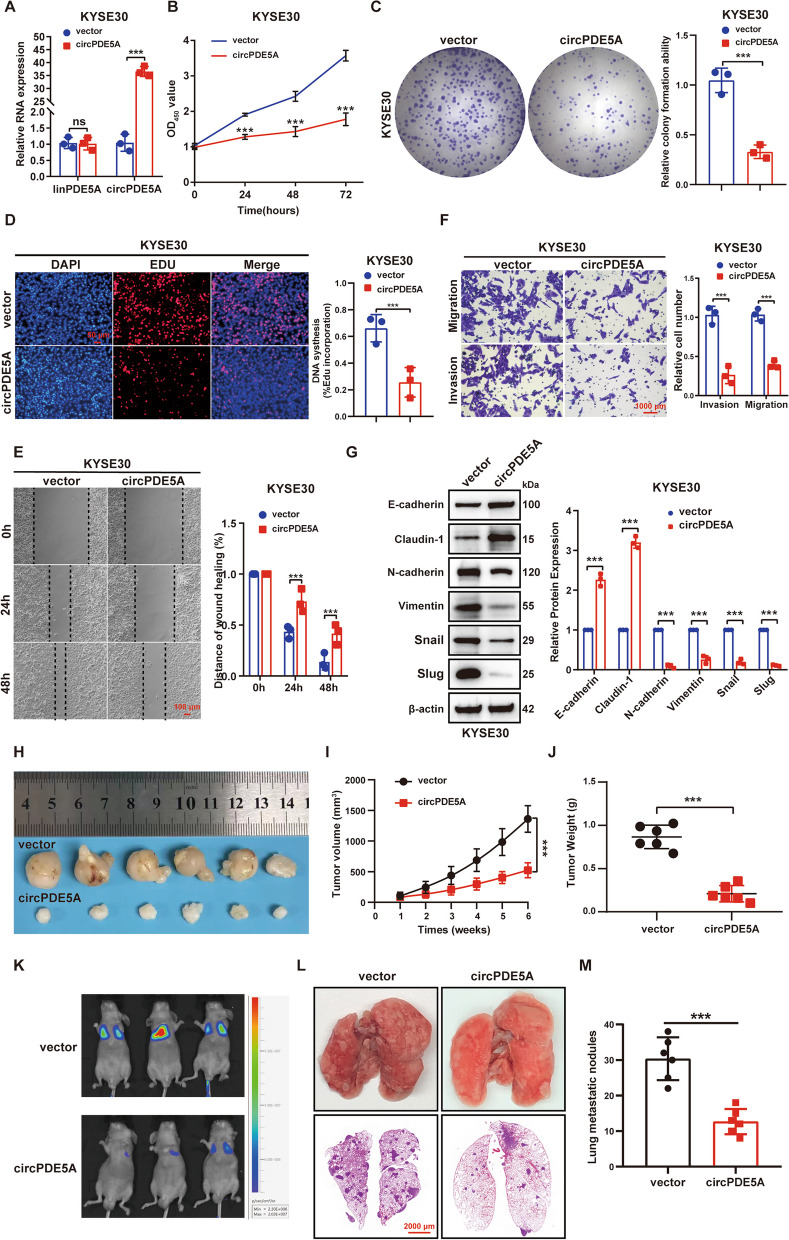
CircPDE5A upregulation inhibits ESCC proliferation and metastasis in vitro and in vivo. **A** RT-qPCR detection of linear PDE5A transcripts and circPDE5A level in KYSE30 cells after treatment with vector and circPDE5A plasmid. **B–D** The proliferative capacity of KYSE30 cells assessed by CCK8 (**B**), plate cloning (**C**), and EdU (**D**) assays after treatment with vector and circPDE5A plasmid. Bar in (**D**) represents 50 μm. **E** Motility of KYSE30 cells assessed by wound healing assay after treatment with vector and circPDE5A plasmid. Bar represents 100 μm. **F** Migration and invasion ability of KYSE30 cells assessed by transwell assay after treatment with vector and circPDE5A plasmid. Bar represents 1,000 μm. **G** WB to detect EMT marker protein levels in KYSE30 cells after treatment with vector and circPDE5A plasmid. **H** Images of nude mouse xenograft tumors (*n* = 6) generated by subcutaneous injection of KYSE30 cells transfected with vector and circPDE5A, respectively. **I** Volume growth curves of nude mouse xenograft tumors. Tumor volume measured every 7 days after inoculation for 6 weeks (*n* = 6, unit: mm^3^). **J** The weight of xenograft tumors in nude mice measured at the end of the experiment (*n* = 6, unit: mg). **K** Representative bioluminescent images of lung metastases formed by tail vein injection of KYSE30 cells transfected with vector and circPDE5A, respectively (*n* = 6). **L** Diagram and HE staining of representative lung tissue from each group. Bar represents 2,000 μm. **M** Number of metastatic lung nodules in each group (*n* = 6). ***P < 0.005. ns, not significant

Based on circPDE5A expression in ESCC cell lines, we selected relatively low-expressing KYSE30 cells for gain-of-function experiments and relatively high-expressing ECA109 cells for loss-of-function experiments (Fig. 1D). We constructed stable circPDE5A-overexpressing KYSE30 cells with normal linear PDE5A expression (Fig. 2A). The cell counting kit-8 (CCK8), plate cloning, 5-ethynyl-2'-deoxyuridine (EdU) cell proliferation, wound healing, transwell, and WB assays showed that circPDE5A overexpression significantly inhibited the proliferation, motility, metastasis, and epithelial-mesenchymal transition (EMT) of ESCC cells (Fig. 2B–G). Moreover, the growth rate of ESCC subcutaneous tumor (Fig. 2H–J) and the number of lung metastatic nodules (Fig. 2K-M) in the circPDE5A overexpression group were significantly lower than those in the control group.

**Fig. 3:**
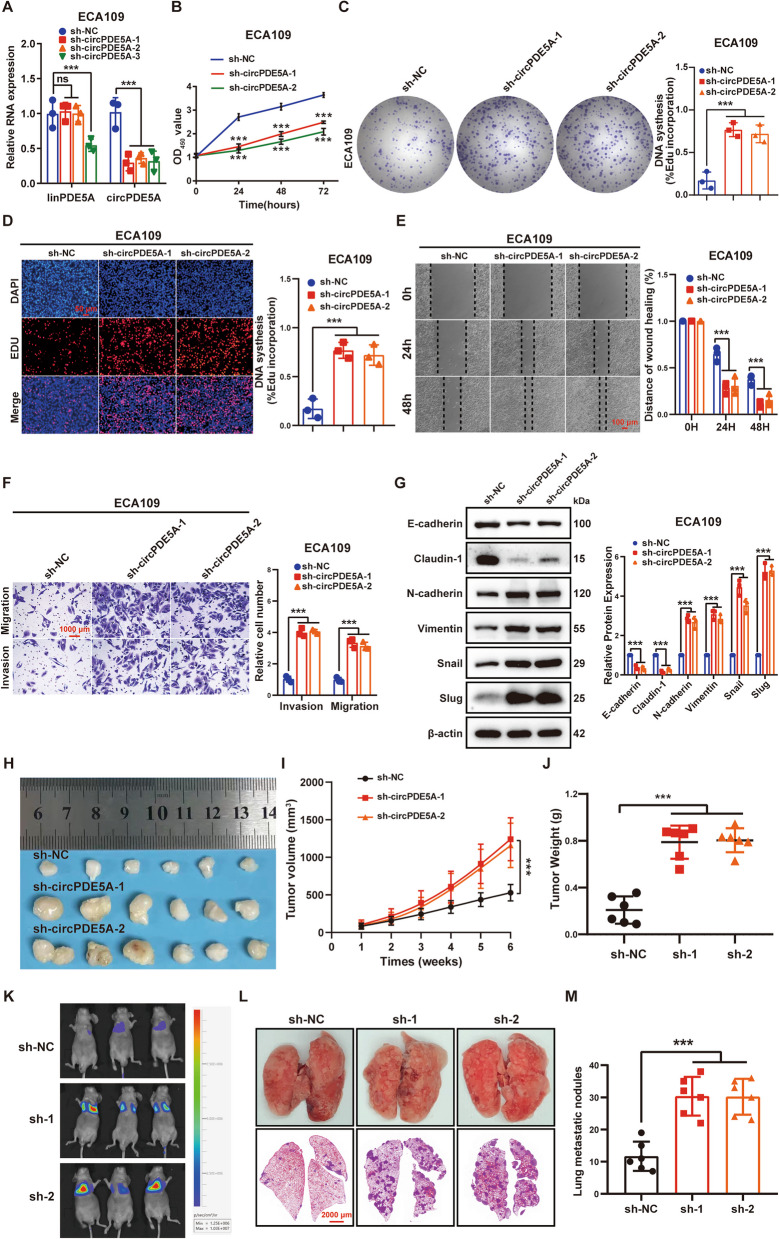
Knockdown of circPDE5A promotes ESCC proliferation and metastasis in vitro and in vivo. **A** RT-qPCR detection of linear PDE5A transcript and circPDE5A level in ECA109 cells after treatment with sh-NC, sh-circPDE5A-1, sh-circPDE5A-2, and sh-circPDE5A-3 plasmid. **B–D** The proliferative capacity of ECA109 cells assessed by CCK8 (**B**), plate cloning (**C**), and EdU (**D**) assays after treatment with sh-NC, sh-circPDE5A-1, and sh-circPDE5A-2 plasmid. Bar in (**D**) represents 50 μm. **E** Motility of ECA109 cells determined by wound healing assay after treatment with sh-NC, sh-circPDE5A-1, and sh-circPDE5A-2 plasmid. Bar represents 100 μm. **F** Migration and invasion ability of ECA109 cells determined by transwell assay after treatment with sh-NC, sh-circPDE5A-1, and sh-circPDE5A-2 plasmid. Bar represents 1,000 μm. **G** EMT marker protein levels in ECA109 cells detected by WB after treatment with sh-NC, sh-circPDE5A-1, and sh-circPDE5A-2 plasmid. **H** Images of xenograft tumors formed in nude mice by subcutaneous injection of ECA109 cells transfected with sh-NC, sh-circPDE5A-1, and sh-circPDE5A-2, respectively. **I** Volume growth curves of nude mouse xenograft tumors. Tumor volume measured every 7 days after inoculation for 6 weeks (*n* = 6, unit: mm^3^). **J** The weight of xenograft tumors in nude mice measured at the end of the experiment (*n* = 6, unit: mg). **K** Representative bioluminescent images of lung metastases formed by tail vein injection of ECA109 cells transfected with sh-NC, sh-circPDE5A-1, and sh-circPDE5A-2, respectively (*n* = 6). **L** Diagram and HE staining of representative lung tissue from each group. Bar represents 2,000 μm. **M** Number of metastatic lung nodules in each group (*n* = 6). ***P < 0.005. ns, not significant

RT-qPCR showed that all three circPDE5A short hairpin RNAs (shRNAs; sh-circPDE5A-1–3) significantly knocked down circPDE5A and that sh-circPDE5A-1 and sh-circPDE5A-2 did not affect linear PDE5A expression (Fig. 3A). Therefore, sh-circPDE5A-1 and sh-circPDE5A-2 were used for the subsequent loss-of-function experiments. In vitro and in vivo experiments showed that circPDE5A knockdown significantly promoted ESCC growth and metastasis (Fig. 3B-M). These results suggest that circPDE5A plays an important role in the regulation of ESCC progression and is a potential target for the treatment of ESCC.

### CircPDE5A encodes a novel protein PDE5A-500aa

**Fig. 4:**
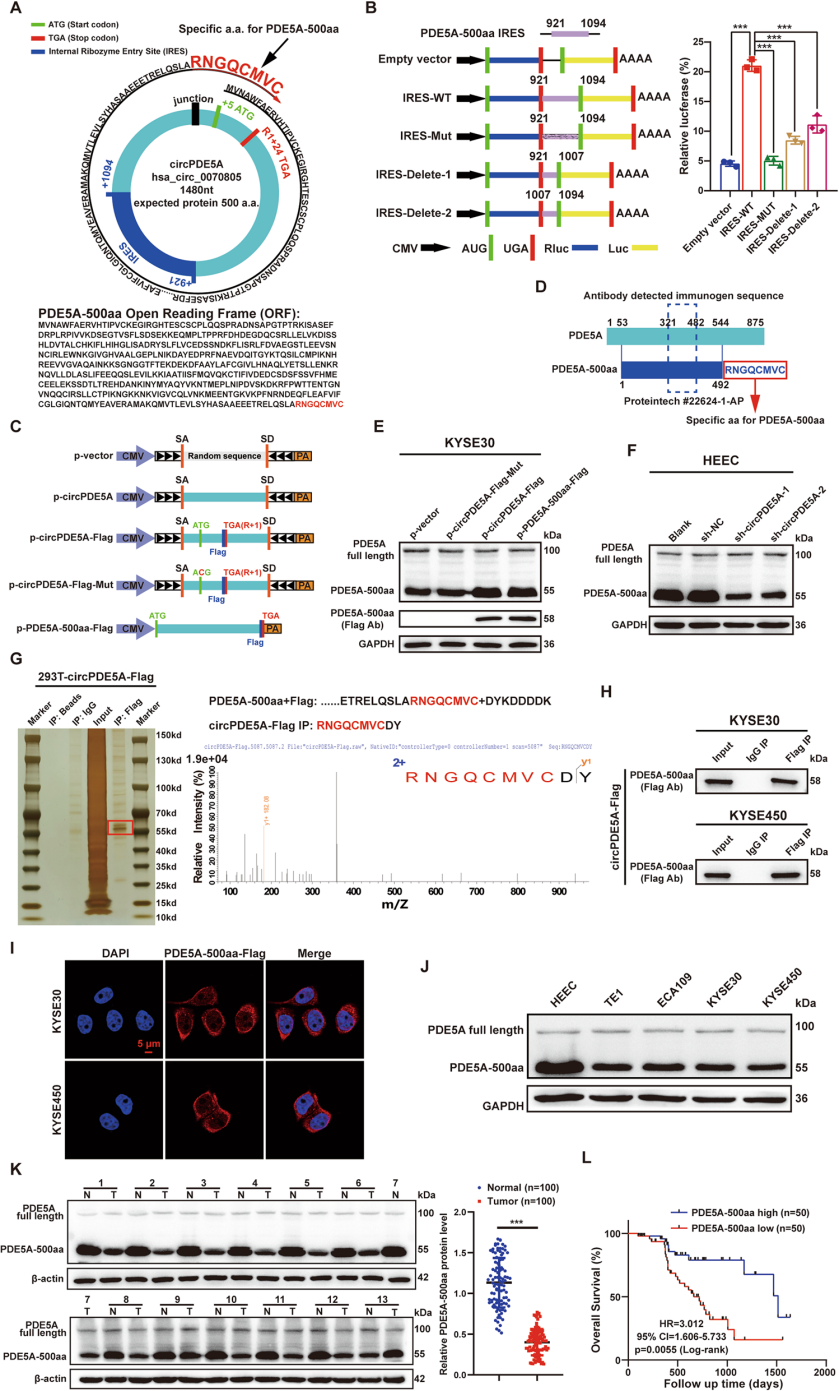
CircPDE5A encodes a novel protein, PDE5A-500aa. **A** Structure of circPDE5A and predicted amino acid sequence of PDE5A-500aa. The upper panel presents a schematic representation of the predicted IRES and ORF sequences in circPDE5A. The lower panel illustrates the predicted amino acid sequence of PDE5A-500aa. **B** Detection of initiator protein translation activity of IRES in circPDE5A. The left panel displays wild-type, truncated, or mutated sequences of IRES in circPDE5A cloned between Rluc and Luc reporter genes with independent initiation codons and termination codons. The right panel depicts the examination of relative luciferase activity of Luc/Rluc in the aforementioned vectors. **C** Three flag-tagged expression vectors were constructed: p-circPDE5A-flag, p-circPDE5A-flag-Mut, and p-PDE5A-500aa to demonstrate the protein-coding function of circPDE5A. **D** The anti-PDE5A antibody (#22,624–1-AP; Proteintech, Wuhan, China) recognizes both PDE5A and PDE5A-500aa. **E** WB for the protein levels of PDE5A, PDE5A-500aa, and flag-tagged PDE5A-500aa in KYSE30 cells treated with various vectors. **F** WB shows the levels of PDE5A and PDE5A-500aa in HEEC cells treated with sh-NC, sh-circPDE5A-1, and sh-circPDE5A-2. **G** The left panel shows IP of anti-flag antibodies in 293 T cells transfected with circPDE5A-flag, and SDS-PAGE separated the pull-down protein. Silver-stained protein bands near 58 kDa are manually excised and analyzed by LC–MS/MS to identify the peptide sequence. The right panel shows the LC–MS/MS identification of the unique peptide sequence of PDE5A-500aa (RNGQCMVC). **H** Co-IP indicates that circPDE5A-flag encodes PDE5A-500aa-flag. **I** Subcellular localization of PDE5A-500aa in KYSE30 and KYSE450 cells detected by immunofluorescence. Bar represents 5 μm. **J** WB to detect the protein levels of PDE5A and PDE5A-500aa in normal esophageal epithelial cells and ESCC cells. **K** WB to detect PDE5A-500aa level in ESCC and adjacent normal tissues. **L** Kaplan–Meier analysis of the correlation between PDE5A-500aa level and OS in patients with ESCC. ***P < 0.005

Predictions from the circBank, TransCirc, and circPrimer databases suggested that the circPDE5A sequence contains an ORF encoding a 500 amino acid (aa) novel protein (referred to as PDE5A-500aa in subsequent mentions) (Fig. 4A). It had a molecular weight of approximately 55 kDa and contained a unique amino acid sequence (RNGQCMVC). To verify the translation initiation activity of the IRES element (from 921–1094 nt) in the circPDE5A sequence (Fig. 4A), we constructed dual-luciferase reporter gene expression vectors for wild-type, mutant, and truncated IRES sequences. The dual-luciferase reporter assay showed that the fluorescence intensity of the mutant and truncated IRES groups was significantly lower than that of the wild-type IRES group (Fig. 4B).

To validate the protein-coding function of circPDE5A, we further constructed three flag-tagged expression vectors, p-circPDE5A-flag, p-circPDE5A-flag-Mut (promoter-mutated), and p-PDE5A-500aa-flag (Fig. 4C). RT-qPCR showed that transfection of the three vectors did not affect the expression of the linear PDE5A transcript, whereas transfection of p-circPDE5A-flag and p-circPDE5A-flag-Mut significantly up-regulated the expression of circPDE5A (Fig. S3A). Furthermore, we found that the anti-PDE5A antibody (Proteintech, Cat. 22,624–1-AP) identified the same immunogenic sequence in PDE5A-500aa as PDE5A (Fig. 4D). WB assay showed that transfection of p-circPDE5A-flag and p-PDE5A-500aa-flag significantly increased the expression of PDE5A-500aa (55 kDa) and PDE5A-500aa-flag (58 kDa), whereas transfection of p-circPDE5A-flag-Mut reversed this effect (Fig. 4E). Knockdown of circPDE5A significantly suppressed PDE5A-500aa expression but did not affect PDE5A expression in a normal ESCC cell line (Fig. 4F). We conducted an immunoprecipitation (IP) assay of circPDE5A-flag-overexpressing 293 T cells using anti-flag antibodies and separated the pull-down proteins by SDS-PAGE. Thereafter, we stained the separated protein bands by silver-staining and excised the 58 kDa band for mass spectrometry (LC–MS/MS) analysis (Fig. 4G). LC–MS/MS analysis identified the specific amino acid sequence (RNGQCMVC) of PDE5A-500aa (Fig. 4G). In addition, the co-immunoprecipitation (Co-IP) assay of the anti-flag antibody pull-down proteins detected the expression of PDE5A-500aa at 58 kDa (Fig. 4H). These results demonstrate that circPDE5A encodes PDE5A-500aa in ESCC cells.

The FISH assay showed that PDE5A-500aa was predominantly distributed in the cytoplasm of ESCC cells (Fig. 4I). WB assay demonstrated that PDE5A-500aa expression was down-regulated in multiple ESCC cell lines (Fig. 4J) and tissues (Fig. 4K). The clinical correlation and KM survival analyses showed that low PDE5A-500aa expression was associated with the later clinicopathological stages (Fig. S3B–D) and poor prognosis (Fig. 4L) of ESCC patients. Furthermore, univariate and multivariate Cox regression analyses showed that low PDE5A-500aa expression was an independent prognostic factor in ESCC patients (Table 2). These results suggest that PDE5A-500aa expression is down-regulated in ESCC and is negatively associated with its poor prognosis.

### CircPDE5A inhibits ESCC proliferation and metastasis in vitro and in vivo by encoding PDE5A-500aa

**Fig. 5:**
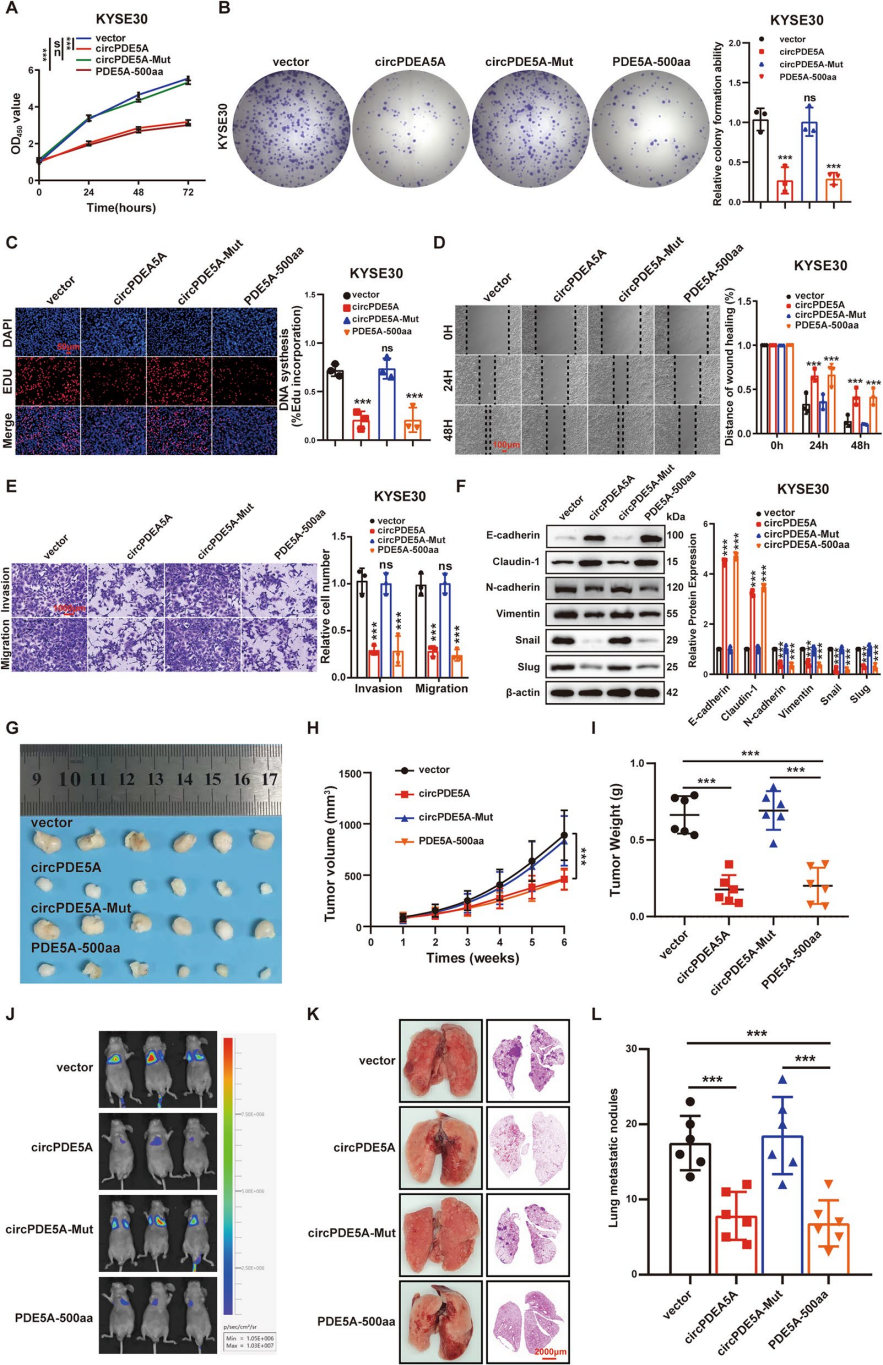
CircPDE5A inhibits ESCC proliferation and metastasis in vitro and in vivo by encoding PDE5A-500aa. **A–C** The proliferative capacity of KYSE30 cells assessed by CCK8 (**A**), plate cloning (**B**), and EdU (**C**) assays following treatment with the vector, circPDE5A, circPDE5A-Mut, and PDE5A-500aa plasmids. Bar in (**C**) represents 50 μm. **D** Motility of KYSE30 cells determined by wound healing assay after treatment with the vector, circPDE5A, circPDE5A-Mut, and PDE5A-500aa plasmids. Bar represents 100 μm. **E** Migration and invasion ability of KYSE30 cells assessed by transwell assay after treatment with the vector, circPDE5A, circPDE5A-Mut, and PDE5A-500aa plasmids. Bar represents 1,000 μm. **F** WB analysis performed to evaluate the levels of EMT marker proteins in KYSE30 cells after treatment with the vector, circPDE5A, circPDE5A-Mut, and PDE5A-500aa plasmids. **G** Representative images of nude mouse xenograft tumors (*n* = 6) generated by subcutaneous injection of KYSE30 cells transfected with the vector, circPDE5A, circPDE5A-Mut, and PDE5A-500aa, respectively. **H** Volume growth curves of nude mouse xenograft tumors. Tumor volume was measured every 7 days after inoculation for 6 weeks (*n* = 6, unit: mm^3^). **I** Measurement of the weight of xenograft tumors in nude mice at the end of the experiment (*n* = 6, unit: mg). **J** Representative bioluminescent images of lung metastases formed by tail vein injection of KYSE30 cells transfected with the vector, circPDE5A, circPDE5A-Mut, and PDE5A-500aa, respectively (*n* = 6). **K** Schematic representation and HE staining of representative lung tissue from each group. Bar represents 2,000 μm. **L** Number of metastatic lung nodules in each group (*n* = 6). ***P < 0.005. ns, not significant

We transfected the three vectors into ESCC cells to explore the biological functions of PDE5A-500aa. The CCK8, plate cloning, EdU cell proliferation, wound healing, transwell, and WB assays showed that transfection of p-circPDE5A and p-PDE5A-500aa vectors inhibited the proliferation, motility, migration, invasion, and EMT of ESCC cells, whereas transfection of p-circPDE5A-Mut reversed the effects caused by transfection with circPDE5A and PDE5A-500aa (Fig. 5A–F**)**. Furthermore, the growth rate of subcutaneous tumors (Fig. 5G–I) and the number of lung metastatic nodules (Fig. 5J–L) were significantly lower in the p-circPDE5A-transfected and p-PDE5A-500aa-transfected groups than in the control group, whereas transfection with p-circPDE5A-Mut reversed the effects caused by transfection with circPDE5A and PDE5A-500aa. These results suggest that circPDE5A inhibits ESCC proliferation and metastasis in vitro and in vivo by encoding PDE5A-500aa.

### PDE5A-500aa interacts with PIK3IP1 and inhibits the PI3K/AKT pathway to suppress ESCC proliferation and metastasis

**Fig. 6:**
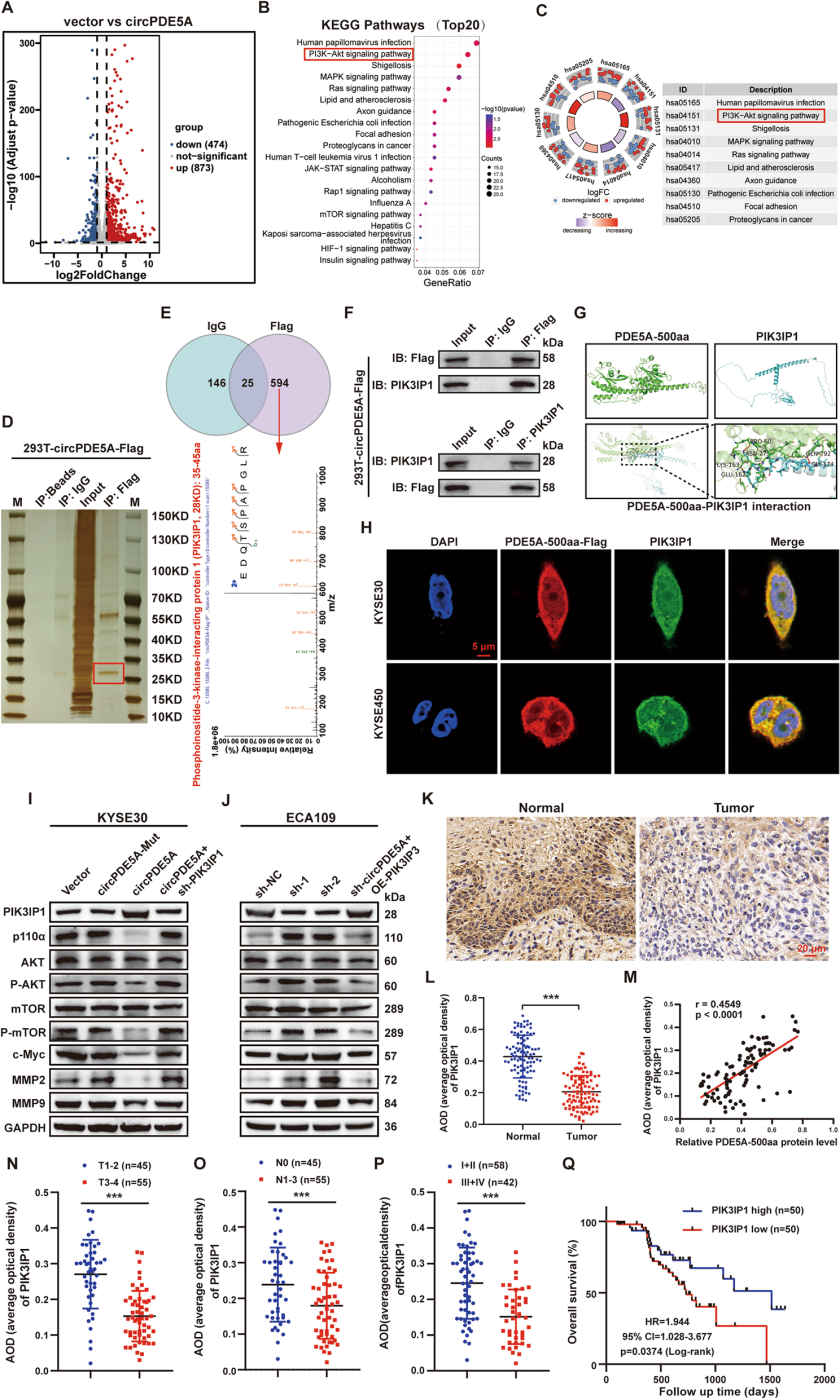
PDE5A-500aa interaction with PIK3IP1 inhibits the PI3K/AKT pathway. **A** Volcano plots comparing fold changes in gene expression in vector and circPDE5A overexpressing KYSE30 cells. **B** Bubble plots of KEGG pathway enrichment analysis showing the top 20 pathways significantly altered in circPDE5A overexpressing KYSE30 cells. **C** Circle plots showing the top 10 significantly altered pathways that were up- or down-regulated in circPDE5A overexpressing KYSE30 cells. **D** Immunoprecipitation (IP) of IgG or anti-flag antibodies in 293 T cells overexpressing circPDE5A-flag, and pull-down proteins visualized by SDS-PAGE and silver staining. **E** Identification of PDE5A-500aa binding proteins by LC–MS/MS analysis. Upper panel: Proteins pulled down by IP with IgG or anti-flag antibodies. LC–MS/MS analysis is used to identify potential proteins specifically binding to PDE5A-500aa. Bottom panel: LC–MS/MS identifies PIK3IP1 protein, a PI3K/AKT pathway regulator, as part of the PDE5A-500aa protein complex. **F** Co-IP shows the interaction between PDE5A-500aa and PIK3IP1. **G** Visualization of docking patterns and key residue interfaces of PDE5A-500aa with PIK3IP1 obtained by PyMOL software. **H** Co-localization of PDE5A-500aa and PIK3IP1 proteins in the cytoplasm of ESCC cells examined by immunofluorescence. Bar represents 5 μm. **I** Levels of PIK3IP1 and PI3K/AKT downstream target proteins in KYSE30 cells detected by WB after treatment with vector, circPDE5A-Mut, circPDE5A, and circPDE5A + si-PIK3IP1 plasmid, respectively. **J** Levels of PIK3IP1 and PI3K/AKT downstream target proteins in ECA109 cells detected by WB after treatment with sh-NC, sh-circPDE5A-1, sh-circPDE5A-2, and sh-circPDE5A + OE-PIK3IP1 plasmid, respectively. **K–L** Immunohistochemical (IHC) detection of PIK3IP1 protein level in ESCC and adjacent normal tissues. Bar represents 20 μm. **M** Correlation analysis between PDE5A-500aa and PIK3IP1 protein level in ESCC tissues. **N–P** IHC detection of PIK3IP1 protein levels in ESCC tissues with different T (**N**), N (**O**), and TNM (**P**) stages. **Q** Kaplan–Meier analysis of the correlation between PIK3IP1 protein level and OS in patients with ESCC. ***P < 0.005

RNA-seq analysis showed that overexpression of circPDE5A up-regulated 873 genes and down-regulated 474 genes in ESCC cells (Fig. 6A). Kyoto Encyclopedia of Genes and Genomes (KEGG) analysis of the differentially expressed genes (DEGs) showed that the PI3K/AKT pathway was significantly enriched and down-regulated (Fig. 6B, C). IP and LC–MS/MS analysis showed that the expression of the protein complex interacting with PDE5A-500aa was significantly enhanced at 25–35 kDa (Fig. 6D), and phosphoinositide-3-kinase interacting protein 1 (PIK3IP1) was identified as the most abundant protein specifically bound to PDE5A-500aa (Fig. 6E and Table S1). Furthermore, the Co-IP assay revealed that PDE5A-500aa interacted with PIK3IP1 in ESCC cells (Fig. 6F). In addition, we predicted the tertiary structure of PDE5A-500aa protein using the I-TASER software and visualized the docking patterns of PDE5A-500aa and PIK3IP1 using the HDOCK and PyMOL software (Fig. 6G). Immunofluorescence (IF) assay confirmed the co-localization of PDE5A-500aa and PIK3IP1 in the cytoplasm of ESCC cells (Fig. 6H). WB assay showed that circPDE5A overexpression significantly increased the expression of PIK3IP1 and suppressed the expression of PI3K/AKT pathway target proteins in ESCC cells, which were reversed by the knockdown of PIK3IP1 (Fig. 6I). In addition, the knockdown of circPDE5A significantly inhibited PIK3IP1 expression and promoted the expression of PI3K/AKT pathway target proteins in ESCC cells, which were reversed by the overexpression of PIK3IP1 (Fig. 6J).

The CCK8, plate cloning, EdU cell proliferation, wound healing, transwell, and WB assays showed that PIK3IP1 knockdown reversed the effects of circPDE5A overexpression on the proliferation, motility, migration, invasion, and EMT of ESCC cells (Fig. S4A-B, and E–H), while PIK3IP1 overexpression reversed the effects of circPDE5A knockdown on the proliferation, motility, migration, invasion and EMT of ESCC cells (Fig. S4C-D, and I-L). The immunohistochemistry (IHC) assay showed that PIK3IP1 was significantly down-regulated in ESCC tissues (Fig. 6K and L) and positively correlated with PDE5A-500aa expression (Fig. 6M). In addition, clinical correlation and KM survival analyses showed that low PIK3IP1 expression was associated with advanced clinicopathological stages (Fig. 6N–P) and poor prognosis (Fig. 6Q) in ESCC. These results suggest that PDE5A-500aa inhibits ESCC proliferation and metastasis by interacting with PIK3IP1 to suppress the PI3K/AKT signaling pathway.

### PDE5A-500aa promotes the de-ubiquitinating activity of PIK3IP1

**Fig. 7:**
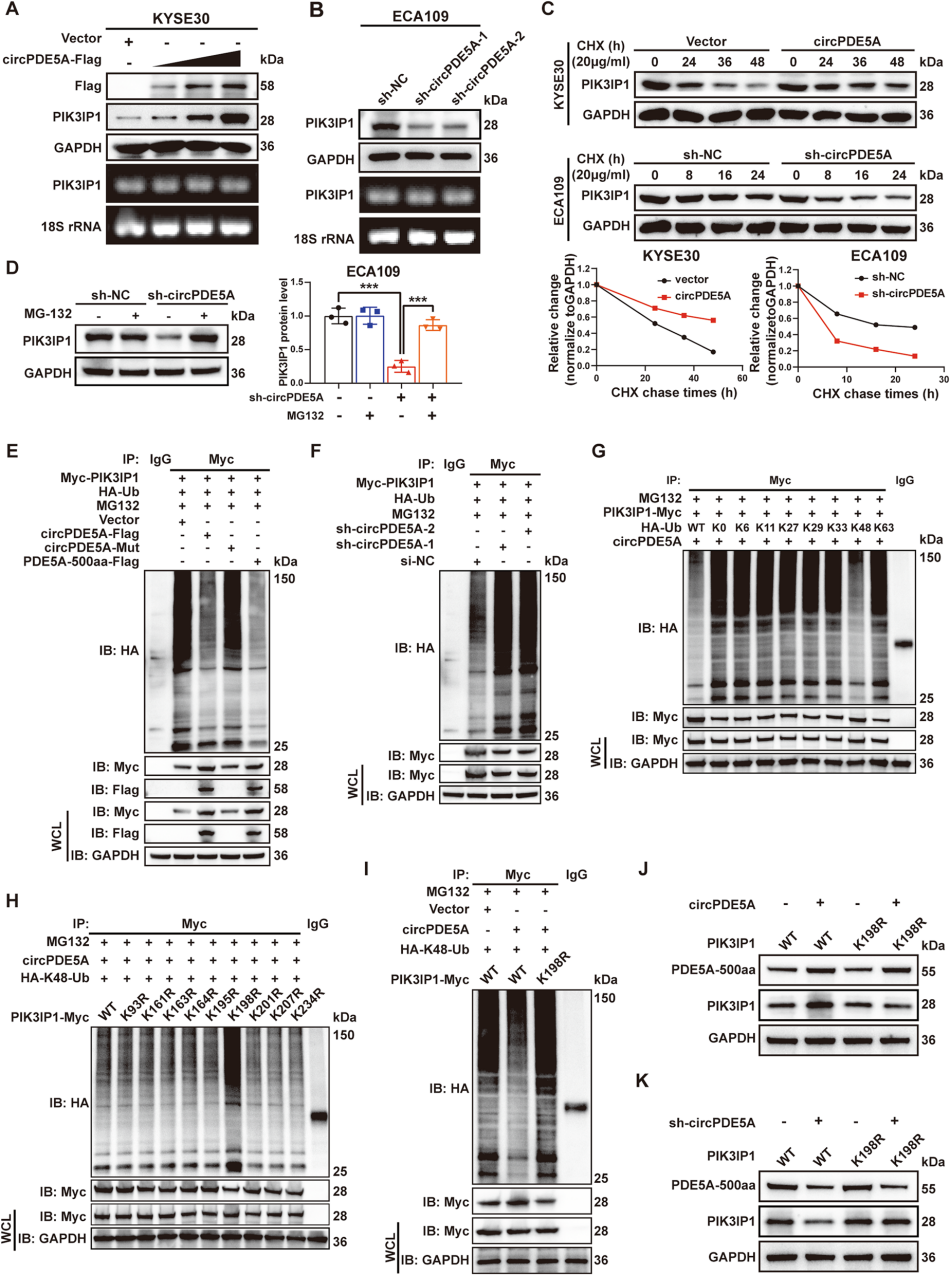
PDE5A-500aa promotes PIK3IP1 de-ubiquitination activity. **A** WB analysis of PDE5A-500aa and PIK3IP1 protein level in KYSE30 cells treated with vector and incremental circPDE5A-flag plasmid. PIK3IP1 mRNA level was assessed by PCR and nucleic acid electrophoresis. **B** WB analysis of PDE5A-500aa and PIK3IP1 protein level in ECA109 cells treated with sh-NC, sh-circPDE5A-1, and sh-circPDE5A-2 plasmids. PIK3IP1 mRNA level was assessed by PCR and nucleic acid electrophoresis. **C** Protein biosynthesis inhibition in KYSE30 or ECA109 cells with 20 μg/mL cycloheximide (CHX). WB analysis of PIK3IP1 protein levels in ESCC cells overexpressing or knocking down circPDE5A at various time points. **D** Inhibition of protein degradation with MG-132 (40 μM) for 8 h. WB analysis of PIK3IP1 protein levels in circPDE5A knockdown ECA109 cells. **E** ESCC cells co-transfected with HA-Ub and Myc-PIK3IP1, treated with MG132. Ubiquitination levels of PIK3IP1 protein detected by IP and WB after treatment with vector, circPDE5A-flag, circPDE5A-Mut, and PDE5A-500aa-flag plasmids. **F** ESCC cells transfected with HA-Ub and Myc-PIK3IP1, treated with MG132. Ubiquitination level of PIK3IP1 protein detected by IP and WB after treatment with sh-NC, sh-PIK3IP1-1, and sh-PIK3IP1-2 plasmids. **G** IP and WB detection of the ubiquitination level of the PIK3IP1 protein in circPDE5A overexpressing ESCC cells co-transfected with HA-tagged different ubiquitin chain vectors with Myc-PIK3IP1 and treated with MG132. **H** ESCC cells co-transfected with HA-K48-Ub and circPDE5A, treated with MG132. Ubiquitination level of PIK3IP1 protein detected by IP and WB after treatment with different Myc-tagged PIK3IP1 lysine site-indicated mutants. **I** ESCC cells transfected with HA-K48-Ub and treated with MG132. Ubiquitination level of PIK3IP1 protein detected by IP and WB after treatment with vector, circPDE5A, and circPDE5A + Myc-PIK3IP1 (K198R) plasmid. **J** Detection of PDE5A-500aa and PIK3IP1 protein level in ESCC cells treated with circPDE5A, PIK3IP1, or PIK3IP1 (K198R) mutant by WB. **K** Detection of PDE5A-500aa and PIK3IP1 protein level in ESCC cells treated with sh-circPDE5A, PIK3IP1, or PIK3IP1 (K198R) mutant by WB. ***P < 0.005

WB and nucleic acid electrophoresis assays showed that PIK3IP1 protein levels were positively associated, while PIK3IP1 mRNA levels were not associated with PDE5A-500aa expression (Fig. 7A, B). Cycloheximide (CHX) assay showed that the half-life of PIK3IP1 was prolonged by circPDE5A overexpression but shortened by circPDE5A knockdown (Fig. 7C). Furthermore, MG132 treatment reversed the effect of circPDE5A knockdown on PIK3IP1 protein expression (Fig. 7D). In vitro ubiquitination assay showed that PIK3IP1 ubiquitination was decreased by circPDE5A and PDE5A-500aa overexpression (Fig. 7E), unaffected by circPDE5A-Mut overexpression (Fig. 7E), and increased by circPDE5A knockdown (Fig. 7F). These results suggest that PDE5A-500aa stabilizes PIK3IP1 levels by promoting its de-ubiquitination.

To determine the type of PDE5A-500aa-mediated PIK3IP1 ubiquitination, we co-transfected ESCC cells with various HA-tagged ubiquitin chain vectors and Myc-PIK3IP1. In vitro ubiquitination assay showed that the k48-associated poly-ubiquitination of PIK3IP1 was attenuated by circPDE5A overexpression (Fig. 7G) but increased by circPDE5A knockdown (Fig. S5A). To further identify the ubiquitination sites of PDE5A-500aa-mediated PIK3IP1 ubiquitination, we constructed nine PIK3IP1 mutants in which distinct lysine residues were mutated to arginine (Fig. S5B). In vitro ubiquitination assay showed that the PIK3IP1 K198R mutant significantly attenuated the effect of circPDE5A overexpression or knockdown on the ubiquitination level of PIK3IP1 (Fig. 7H and Fig. S5C). In addition, the PIK3IP1 K198R mutant reversed the alterations in PIK3IP1 ubiquitination level (Fig. 7I and Fig. S5D) and PIK3IP1 protein expression (Fig. 7J, K) caused by circPDE5A overexpression or knockdown. These results suggest that PDE5A-500aa stabilizes PIK3IP1 levels by promoting its de-ubiquitination at the K198 residue on the k48-linked poly-ubiquitin chain.

### PDE5A-500aa promotes USP14-mediated de-ubiquitination of PIK3IP1

**Fig. 8:**
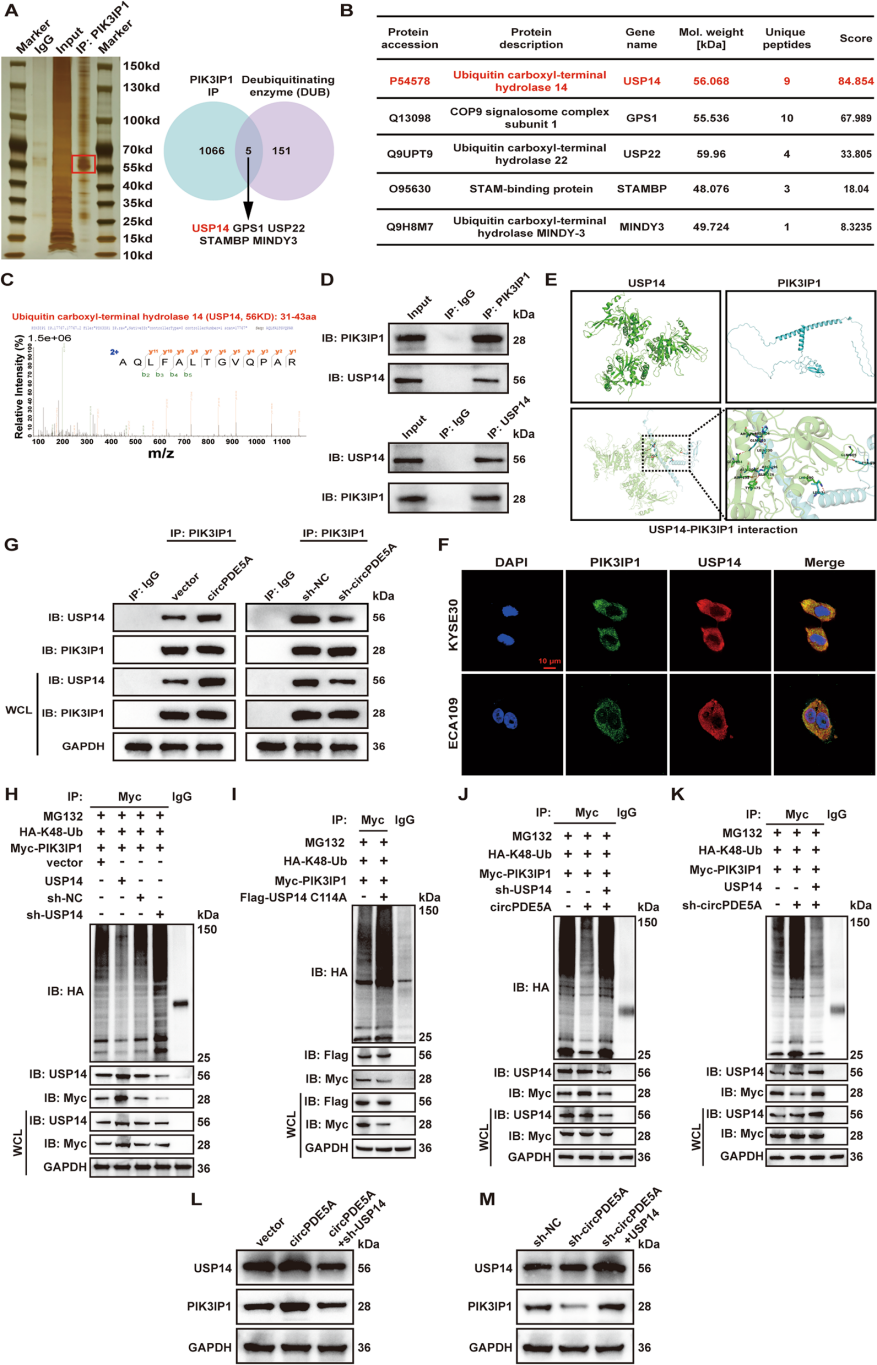
PDE5A-500aa stabilizes PIK3IP1 via USP14-mediated de-ubiquitination. **A** Investigation of PIK3IP1 interacting proteins and de-ubiquitinating enzymes. Left panel: Immunoprecipitation (IP) of whole cell lysates from PIK3IP1 overexpressing 293 T cells with anti-PIK3IP1 antibody, followed by visualization of the pull-down proteins by WB and silver staining. Right panel: Venn diagram illustrating de-ubiquitinating enzymes potentially bound to PIK3IP1 identified by LC–MS/MS. **B** Specific information on the five de-ubiquitinating enzymes identified by LC–MS/MS, ranked in order of enriched abundance. **C** Secondary mass spectra of the USP14 protein identified by LC–MS/MS. **D** Validation of the interaction between PIK3IP1 and USP14 in ESCC cells by Co-IP. **E** Visualization of docking patterns and key residue interfaces for protein–protein interaction between PIK3IP1 and USP14, obtained using PyMOL software. **F** Immunofluorescence analysis showing co-localization of PIK3IP1 and USP14 proteins in the cytoplasm of ESCC cells. Bar represents 10 μm. **G** IP and WB to detect the interaction between PIK3IP1 and USP14 in circPDE5A overexpressing and knockdown ESCC cells. **H** ESCC cells transfected with HA-K48-Ub and Myc-PIK3IP1, treated with MG132. Detection of PIK3IP1 ubiquitination levels in USP14 overexpressing or knockdown ESCC cells using IP and WB. **I** ESCC cells transfected with HA-K48-Ub and Myc-PIK3IP1, treated with MG132. Detection of PIK3IP1 ubiquitination level after treatment with the flag-tagged USP14 indicated mutant (flag-USP14-C114A) using IP and WB. **J** ESCC cells transfected with HA-K48-Ub and Myc-PIK3IP1, treated with MG132. Detection of PIK3IP1 ubiquitination level after treatment with circPDE5A or sh-USP14 plasmid using IP and WB. **K** ESCC cells transfected with HA-K48-Ub and Myc-PIK3IP1, treated with MG132. Detection of PIK3IP1 ubiquitination level after treatment with sh-circPDE5A or USP14 plasmid using IP and WB. **L** WB analysis of USP14 and PIK3IP1 level in ESCC cells after treatment with circPDE5A or sh-USP14 plasmid. **M** WB analysis of USP14 and PIK3IP1 level in ESCC cells after treatment with sh-circPDE5A or USP14 plasmid

LC–MS/MS analysis of IP pull-down proteins using anti-PIK3IP1 antibodies revealed that the potential PIK3IP1-interacting proteins included five de-ubiquitinating enzymes, among which the ubiquitin-specific protease 14 (USP14) protein had the highest abundance in ESCC cells (Fig. 8A–C). The Co-IP assay further verified that PIK3IP1 interacted with USP14 in ESCC cells (Fig. 8D). The docking patterns of PIK3IP1 and USP14 were visualized using the HDOCK and PyMOL software (Fig. 8E). IF assay showed that PIK3IP1 and USP14 were co-localized in the cytoplasm of ESCC cells (Fig. 8F). Furthermore, the Co-IP assay showed that the interaction between PIK3IP1 and USP14 was significantly promoted by circPDE5A overexpression but significantly attenuated by circPDE5A knockdown (Fig. 8G).

In vitro ubiquitination assay showed that the level of PIK3IP1 ubiquitination was decreased by USP14 overexpression and increased by USP14 knockdown (Fig. 8H). The catalytic cysteine (Cys114) has been reported to be an important de-ubiquitinating enzyme active site of USP14 [24, 25]. Therefore, we further constructed an active site mutant of USP14 (USP14 C114A) to verify the role of this site in the regulation of PIK3IP1. The result showed that the USP14 mutant significantly increased the level of ubiquitination of PIK3IP1 (Fig. 8I). The Co-IP showed that USP14 knockdown reversed the circPDE5A overexpression-mediated changes in PIK3IP1 ubiquitination levels (Fig. 8J) and protein expression (Fig. 8L), while USP14 overexpression reversed the circPDE5A knockdown-mediated changes in PIK3IP1 ubiquitination levels (Fig. 8K) and protein expression (Fig. 8M). These results suggest that PDE5A-500aa stabilizes PIK3IP1 levels by promoting USP14-mediated de-ubiquitination of the PIK3IP1 k48-linked poly-ubiquitin chain.

### NPs-mediated overexpression of circPDE5A and PDE5A-500aa inhibits ESCC cell proliferation and metastasis in vitro

**Fig. 9:**
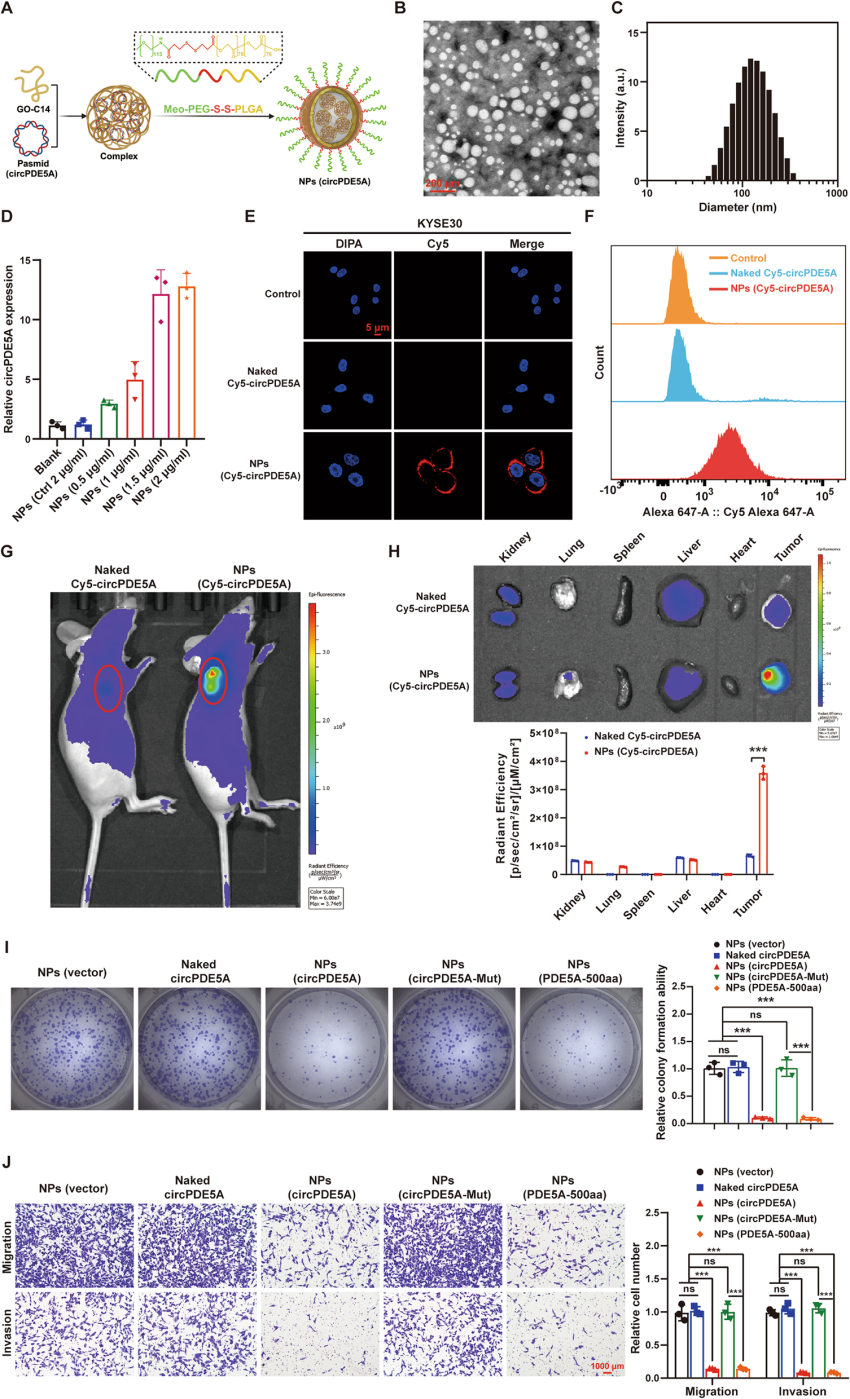
NPs-mediated upregulation of circPDE5A and PDE5A-500aa inhibits ESCC cell proliferation and metastasis in vitro. **A** Schematic illustration of the reduction-responsive circRNA nanoplatforms made with the cationic lipid-like compound G0-C14 and amphiphilic copolymer Meo-PEG*-S–S-*PLGA. **B–C** Transmission electron microscope image (**B**) and size distribution (**C**) of NPs (circPDE5A) in aqueous solution. Bar in (**B**) represents 200 μm. **D** RT-qPCR analysis of the relative level of circPDE5A after treatment of ESCC cells with different doses of NPs (circPDE5A). **E** Confocal fluorescence microscopy to detect the uptake of Cy5-circRNA by ESCC cells in response to different treatment groups. Bar represents 5 μm. **F** Flow cytometric analysis of Cy5-circRNA uptake by ESCC cells in response to different treatment groups. **G** Fluorescence imaging of the ESCC subcutaneous tumor model in nude mice after tail vein injection of naked Cy5-circPDE5A plasmid and NPs (Cy5-circPDE5A). **H** Fluorescence imaging of isolated major organs and tumors in nude mice after tail vein injection of naked Cy5-circPDE5A plasmid and NPs (Cy5-circPDE5A). **I** The proliferative capacity of ESCC cells was detected by plate cloning assay after treatment with NPs (vector), naked circPDE5A plasmid, NPs (circPDE5A), NPs (circPDE5A-Mut), and NPs (PDE5A-500aa). **J** Migration and invasion ability of ESCC cells determined by transwell assay after treatment with NPs (vector), naked circPDE5A plasmid, NPs (circPDE5A), NPs (circPDE5A-Mut), and NPs (PDE5A-500aa). Bar represents 1,000 μm. ***P < 0.005. ns, not significant

We constructed circRNA-loaded nanoparticles by combining methoxyl-poly(ethylene glycol)-b-poly(lactic-co-glycolic acid) copolymer with a reduction-responsive disulfide linker (Meo-PEG-*S–S-*PLGA) and an amphiphilic cationic lipid compound (G0-C14) Fig. S6A) that we developed previously [23] (Fig. 9A). The circPDE5A-NPs were spherical (Fig. 9B) with a circRNA encapsulation efficiency of approximately 80%, an average size of approximately 100 nm (Fig. 9C), and a zeta point of -15.9 mV (Fig. S6B). RT-qPCR showed that the circPDE5A–NPs could up-regulate circPDE5A expression in ESCC cells in a dose-dependent manner (Fig. 9D). Laser confocal scanning (Fig. 9E) and flow cytometry (Fig. 9F) showed that the uptake of Cy5-circPDE5A-NPs by KYSE30 cells was significantly higher than that of the control and naked Cy5-circPDE5A plasmids. Fluorescence in vivo imaging showed that the Cy5 fluorescence signal was highly accumulated at the tumor site (Fig. 9G) and its fluorescence intensity was significantly higher in the Cy5-circPDE5A-NP group compared to the naked Cy5-circPDE5A group (Fig. 9H).

The CCK8 (Fig. S6C), plate cloning (Fig. 9I), and transwell (Fig. 9J) assays showed that circPDE5A-NPs and PDE5A-500aa-NPs significantly inhibited the proliferation, migration, and invasion of ESCC cells. These results suggest that NP-mediated overexpression of circPDE5A and PDE5A-500aa inhibits ESCC proliferation and metastasis in vitro.

### NPs-mediated overexpression of circPDE5A and PDE5A-500aa inhibits ESCC proliferation and metastasis in vivo

**Fig. 10:**
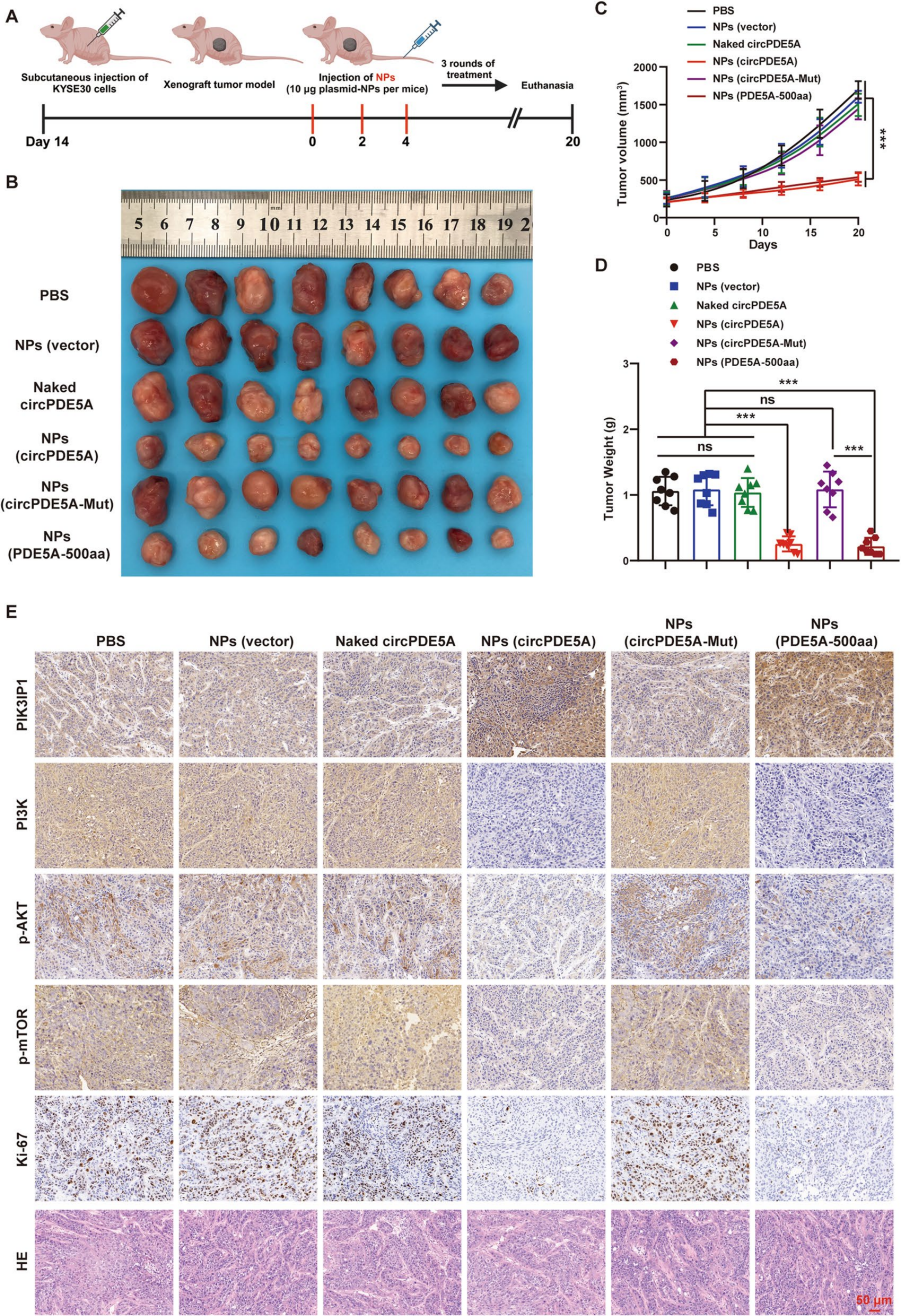
NPs-mediated upregulation of circPDE5A and PDE5A-500aa inhibits ESCC proliferation in vivo. **A** Schematic representation of a subcutaneous xenograft tumor model treated with NPs. **B** Photographs of subcutaneous xenograft tumors collected from mice at the end of treatment in each group (*n* = 8). **C** Volume growth curves of subcutaneous xenograft tumors in each treatment group after starting treatment. Tumor volume was measured every 4 days after the start of treatment (*n* = 8, unit: mm^3^). **D** The weight of subcutaneous xenograft tumors in each treatment group measured at the end of the experiment (*n* = 8, unit: mg). **E** IHC staining for PIK3IP1, PI3K, p-AKT, p-mTOR, and Ki67 in tumor tissues after systemic treatment in subcutaneous xenograft tumor nude mice with ESCC in each group. Bar represents 50 μm

We assessed the effect of NPs on ESCC growth in vivo. After 2 weeks post subcutaneous injection of KYSE30 cells (to establish a subcutaneous xenograft tumor model), we divided 48 nude mice into six groups (*n* = 8/group) and subjected them to different treatments. The mice were injected via the tail vein with PBS, vector-NPs naked circPDE5A plasmids, circPDE5A-NPs, circPDE5A-Mut-NPs, and PDE5A-500aa-NPs. The detailed treatment course of the subcutaneous xenograft tumor model is shown in Fig. 10A. The tumor size, growth rate, and weight of the circPDE5A-NP and PDE5A-500aa-NP treatment groups were significantly smaller than in the control group, while treatment with circPDE5A-Mut-NPs reversed the effects of treatment with circPDE5A-NPs and PDE5A-500aa-NPs (Fig. 10B–D). Notably, there was no significant difference in the body weights of the mice in each group during the treatment period (Fig. S7). HE staining showed no significant histological changes in the major organ tissues of each group (Fig. S8), and routine blood analyses showed that the major parameters of liver and kidney function were within the normal range for all the groups (Fig. S9). These results suggest that the in vivo toxicity of circRNA-NPs is low. Unsurprisingly, treatment with circPDE5A-NPs and PDE5A-500aa-NPs significantly increased protein expression of PDE5A-500aa in subcutaneous tumors, whereas treatment with circPDE5A-Mut-NPs reversed this effect (Fig. S10A). IHC of subcutaneous tumors showed that the expression of PIK3IP1 was significantly up-regulated and the expression of PI3K/AKT pathway target proteins (PI3K and p-AKT) and Ki67 were significantly down-regulated in the circPDE5A-NP and PDE5A-500aa-NP treatment groups, whereas treatment with circPDE5A-Mut-NPs reversed the effects induced by treatment with circPDE5A-NPs and PDE5A-500aa-NPs (Fig. 10E). These results suggest that NP-mediated overexpression of circPDE5A and PDE5A-500aa inhibits ESCC growth in vivo by suppressing the PI3K/AKT pathway.

**Fig. 11:**
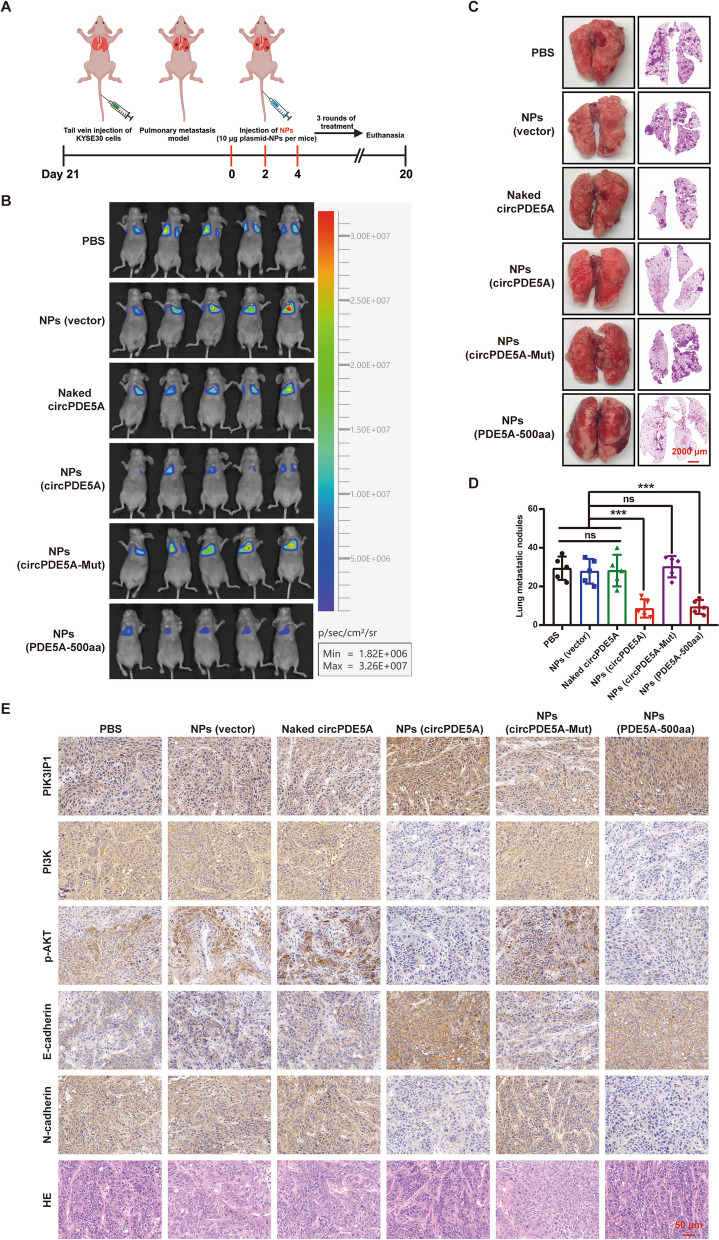
NPs-mediated upregulation of circPDE5A and PDE5A-500aa inhibits ESCC metastasis in vivo. **A** Schematic representation of a lung metastasis model treated with NPs. **B** Bioluminescence images of mice in each group at the end of the experiment (*n* = 5). **C** Schematic diagram and HE staining of representative lung tissue from each group. Bar represents 2,000 μm. **D** Number of metastatic lung nodules in each group (*n* = 5). **E** IHC staining for PIK3IP1, PI3K, p-AKT, E-cadherin, and N-cadherin in tumor tissues after systemic treatment in subcutaneous xenograft tumor nude mice with ESCC in each group. Bar represents 50 μm. ***P < 0.005. ns, not significant

Next, we evaluated the effect of NPs on ESCC metastasis in vivo. After 3 weeks of tail vein injection of KYSE30 cells (to establish a lung metastasis model), we divided 30 nude mice into six groups (*n* = 5/group). The detailed treatment course of the lung metastasis model is shown in Fig. 11A. The results showed that the number of lung metastatic nodules in the circPDE5A–NP and PDE5A-500aa–NP treatment groups was significantly less than that in the control group, while treatment with circPDE5A-Mut-NPs reversed the effects of treatment with circPDE5A-NPs and PDE5A-500aa-NPs (Fig. 11B–D). WB assay showed that treatment with circPDE5A-NPs and PDE5A-500aa-NPs significantly increased protein expression of PDE5A-500aa in lung metastases, whereas treatment with circPDE5A-Mut-NPs reversed this effect (Fig. S10B). IHC of lung metastases showed that the expression of PIK3IP1 was significantly up-regulated and the expression of PI3K/AKT pathway target proteins and EMT phenotype were significantly down-regulated in the circPDE5A-NP and PDE5A-500aa-NP treatment groups, whereas treatment with circPDE5A-Mut-NPs reversed the effects induced by treatment with circPDE5A-NPs and PDE5A-500aa-NPs (Fig. 11E). These results suggest that NP-mediated overexpression of circPDE5A and PDE5A-500aa inhibits ESCC metastasis in vivo by suppressing the PI3K/AKT pathway.

## Discussion

The PI3K/AKT signaling pathway plays an important role in the development and progression of ESCC, and its hyperactivation is associated with the later clinicopathological stages and poor prognosis of ESCC patients [16]. Most functional circRNAs can act as upstream regulators of classical signaling pathways to affect tumor cell growth, metastasis, and drug resistance [6]. Studies have shown that protein-coding circRNAs have important biological functions in ESCC progression [11, 26]. However, the protein-coding circRNAs involved in the regulation of the PI3K/AKT signaling pathway are still unknown.

Phosphodiesterase 5A (PDE5A), a member of the cyclic nucleotide phosphodiesterase family, has been shown to play an important role in promoting the progression of thyroid cancer [27] and melanoma [28]. A recent study showed that hsa_circ_0002474, one of the cyclic transcripts of PDE5A, is downregulated in prostate cancer tissues and inhibits prostate metastasis via the circPDE5A-WTAP-EIF3C-MAPK pathway [29]. However, the role of circular transcripts of PDE5A in ESCC remains to be elucidated. In this study, we found that circPDE5A was significantly down-regulated in ESCC and was significantly negatively associated with its poor prognosis. Furthermore, we found that circPDE5A-encoded PDE5A-500aa interacts with PIK3IP1 and promotes its USP14-mediated de-ubiquitination, thereby attenuating PI3K/AKT signaling and inhibiting ESCC progression. Therefore, our results indicate that circPDE5A is an effective therapeutic target for ESCC.

PI3Ks are key molecules in the PI3K/AKT pathway, among which class I PI3Ks, containing the p110 catalytic subunit and the p85 regulatory subunit, are essential in cancer [30–32]. The p85 and p110 activating mutations and amplifications are associated with the development and progression of various tumors [33, 34]. The lipid phosphatases SHIP, inositol polyphosphate-4-phosphatase type II (INPP4B), and phosphatase and tensin homolog (PTEN) were found to negatively regulate the PI3K/AKT pathway [35]. PIK3IP1 is a recently discovered negative regulator of PI3K, which can bind p110 and prevent its activation by p85, thereby down-regulating PI3K signaling to inhibit tumor growth [36, 37]. Recent studies suggest that PIK3IP1 may act as a tumor suppressor to regulate tumor cell function [37] and anti-tumor immune activity [38, 39]. In addition, PIK3IP1 is highly expressed in PI3Kα inhibitor-sensitive gastric cancer cells and can be used as a marker to detect the anti-cancer activity of PI3Kα inhibitor [40]. However, the role of PIK3IP1 in ESCC is not fully understood. This study showed that the PDE5A-500aa interacts with PIK3IP1 and induces its de-ubiquitination, thereby attenuating PI3K/AKT signaling in ESCC.

USP14 is a proteasome-associated de-ubiquitinating enzyme that protects protein substrates from degradation by removing the ubiquitin chain from the proteasome-bound substrate [41]. The post-translational modification process induced by the dysregulation of USP14 is involved in the onset and progression of tumors, immune responses, and viral infections through the modulation of multiple signaling pathways [25, 42, 43]. For instance, USP14 interacts with ceramide synthase 1 (CERS1) to inhibit the progression of brain metastasis by down-regulating the PI3K/AKT/mTOR signaling pathway [44]. High expression of USP14 is associated with poor prognosis of ESCC patients [45], and its knockdown inhibits ESCC cell proliferation [46]. Our study showed that PDE5A-500aa overexpression increased PIK3IP1 protein expression without affecting PIK3IP1 mRNA expression. In addition, PIK3IP1 expression was positively correlated with PDE5A-500aa expression in ESCC tissues. Further analysis showed that PDE5A-500aa interacts with PIK3IP1 to induce USP14-mediated de-ubiquitination of the k48-linked polyubiquitin chain at its K198 residue.

In recent years, circRNAs have been used for the development of vaccines against SARS-CoV-2 [47] and hard-to-treat malignancies [48], owing to their highly stable and continuously translatable properties. A recent study showed that administration of vaccines consisting of tumor-specific circRNA or its encoded peptides in mice bearing breast cancer tumors or melanoma induced enhanced infiltration of tumor-antigen-specific cytotoxic T cells, which led to effective tumor control [10]. In addition, functional circRNAs are widely used in preclinical studies to develop new therapeutic targets for tumors [6]. For instance, Guan et al. [49] developed chitosan-epigallocatechin gallate nanoparticle-based nanomedicines that could deliver circSPIRE1 overexpression plasmid to tumor cells, thus successfully inhibiting renal cell carcinoma metastasis in vivo. However, circRNA-based therapeutic strategies have not been reported for ESCC. In this study, we constructed a Meo-PEG*-S–S-*PLGA-based reduction-responsive nanoplatforms capable of delivering circPDE5A and PDE5A-500aa overexpression plasmids to tumor cells, which can successfully inhibit ESCC growth and metastasis in vivo.

Currently, there are over 50 new drugs that can inhibit the PI3K/AKT/mTOR pathway in various stages of development [50]. However, inhibitors of this pathway are clinically ineffective for several reasons. With the rapid development of RNA-related technologies, novel RNA-based therapies have received unprecedented attention in recent years. In this study, we found that circPDE5A inhibits the PI3K/AKT signaling pathway by encoding PDE5A-500aa, thereby inhibiting ESCC progression, indicating that circPDE5A may serve as novel a therapeutic target for ESCC treatment.

## Conclusion

Our study demonstrates that circPDE5A inhibits ESCC proliferation and metastasis in vitro and in vivo by encoding PDE5A-500aa. Further analysis revealed that PDE5A-500aa promoted USP14-mediated de-ubiquitination-associated post-translational modification of PIK3IP1 to attenuate PI3K/AKT signaling, thereby inhibiting ESCC proliferation and metastasis. Moreover, we found that nanoplatforms loaded with circPDE5A and PDE5A-500aa overexpression plasmids successfully inhibited ESCC growth and metastasis in vitro and in vivo. Therefore, our findings provide a basis for the development of potential circPDE5A-based therapeutic targets for ESCC.

### Supplementary Information

**Supplementary Material 1.**

## Data Availability

The data generated in this study are publicly available in Gene Expression Omnibus (GEO) at GSE250413 and GSE250323. All data relevant to the study are included in the article or provided as supplementary information. All data and sources associated with this study are available from the corresponding author upon reasonable request.

## Abbreviations

EC: Esophageal cancer
ESCC: Esophageal squamous cell carcinoma
circRNAs: Circular RNAs
miRNA: MicroRNA
m6A: N6-methyladenosine
IRES: Internal ribosomal entry sites
ORFs: Open reading frames
PI3K: Phosphatidylinositol 3-kinase
RT-qPCR: Reverse transcription-quantitative polymerase chain reaction
FISH: Fluorescence in situ hybridization
IHC: Immunohistochemistry
WB: Western blot
DMF: Dimethylformamide
DLS: Dynamic light scattering
TEM: Transmission electron microscopy
EE: Encapsulation efficiency
DMSO: Dimethyl sulfoxide
HE: Hematoxylin and eosin
GEO: Gene Expression Omnibus
OS: Overall survival
PFS: Progression-free survival
KM: Kaplan–Meier
PDE5A: Phosphodiesterase 5A
cDNA: Complementary DNA
gDNA: Genomic DNA
CCK8: Cell counting kit-8
EdU: 5-Ethynyl-2'-deoxyuridine
EMT: Epithelial-mesenchymal transition
shRNAs: Short hairpin RNAs
aa: Amino acid
IP: Immunoprecipitation
LC–MS/MS: Liquid chromatograph mass spectrometer
KEGG: Kyoto Encyclopedia of Genes and Genomes
DEGs: Differentially expressed genes
PIK3IP1: Phosphoinositide-3-kinase interacting protein 1
IF: Immunofluorescence
IHC: Immunohistochemistry
CHX: Cycloheximide
INPP4B: Inositol polyphosphate-4-phosphatase type II
PTEN: Phosphatase and tensin homolog
USP14: Ubiquitin-specific protease 14
CERS1: Ceramide synthase 1
