# Supplementary material for: CircPDE5A-encoded novel regulator of the PI3K/AKT pathway inhibits esophageal squamous cell carcinoma progression by promoting USP14-mediated de-ubiquitination of PIK3IP1
Source: J Exp Clin Cancer Res. 2024 Apr 24;43:124. doi: 10.1186/s13046-024-03054-3 (PMC11040784; doi:10.1186/s13046-024-03054-3)
Supplement: Supplementary file 1 — Supplementary Material 1. [file 13046_2024_3054_MOESM1_ESM.docx]

**CircPDE5A-encoded novel regulator of the PI3K/AKT pathway inhibits esophageal squamous cell carcinoma progression by promoting USP14-mediated de-ubiquitination of PIK3IP1**

Kai Lei,^#1,2^ Ruihao Liang,^#1,2^ Jialu Liang,^#1,2^ Nan Lu,^#1,3^ Jing Huang,^1,2^ Ke Xu,^1,2^ Binghua Tan,^1,2^ Kexi Wang,^1,2^ Yicheng Liang,^1,2^ Wenjian Wang,^1,2^ Huayue Lin,^1,4^* and Minghui Wang^1,2^*

**Affiliations**

^1^Guangdong Provincial Key Laboratory of Malignant Tumor Epigenetics and Gene Regulation, Sun Yat-sen Memorial Hospital, Sun Yat-sen University, Guangzhou 510120, China

^2^Department of Thoracic Surgery, Sun Yat-sen Memorial Hospital, Sun Yat-sen University, Guangzhou 510120, China

^3^Nanhai Translational Innovation Center of Precision Immunology, Sun Yat-sen Memorial Hospital, Foshan 528200, China

^4^Breast Tumor Center, Sun Yat-sen Memorial Hospital, Sun Yat-sen University, Guangzhou 510120, China

^#^ These authors have contributed equally to this work and share the first authorship

*** Corresponding authors:**

Prof. Lin Huayue, Breast Tumor Center, Sun Yat-sen Memorial Hospital, Sun Yat-sen University, Guangzhou 510120, China. E-mail: linhy29@mail.sysu.edu.cn

Prof. Wang Minghui, Department of Thoracic Surgery, Sun Yat-sen Memorial Hospital, Sun Yat-sen University, Guangzhou 510120, China. E-mail: wmingh@mail.sysu.edu.cn

**Supplementary methods**

**1. Total RNA extraction and RT-qPCR**

Samples were subjected to total RNA extraction using Trizol reagent (Invitrogen, Carlsbad, CA, USA) following the manufacturer's instructions. Subsequently, the concentration and purity of all RNA samples were measured using NanoDrop2000 (Thermo Scientific, Wilmington, DE, USA). The corresponding cDNA was synthesized using HiScript II Q RT SuperMix for qPCR (Vazyme, Nanjing, China).

For qPCR, reaction systems were prepared with AceQ qPCR SYBR Green Master Mix (Vazyme). The qPCR reactions were carried out on the LightCycler®96 system (Roche, Basel, Switzerland). The RT-qPCR reaction process included predenaturation (1 cycle), amplification (40 cycles), and melting (1 cycle). The relative expression of the genes was calculated using the 2^−ΔΔCT^ method. The primer sequences for all RT-qPCR reactions are provided in **Table S2**.

**2. Nucleic acid electrophoresis and Sanger sequencing**

Total RNA was extracted from KYSE30 cells using Trizol reagent (Invitrogen) following the manufacturer's instructions. Subsequently, cDNA was synthesized using HiScript II Q RT SuperMix for qPCR (Vazyme). PCR reactions were performed using 2 × Phanta Flash Master Mix (Vazyme) with specific circPDE5A primers (both divergent and convergent).

For analysis, the PCR products amplified with different circPDE5A primers were separated by electrophoresis on a 3% agarose gel. Simultaneously, to confirm the accurate sequence of the reverse splice site of circPDE5A, the PCR product from the divergent primer was subjected to Sanger sequencing, employing the sequencing primer 5′-AGGGGCACTGTTATCTGCAC-3′. Individual clones were sequenced using Applied Biosystems BigDye terminator mix v3.1.

To distinguish between true A-to-G conversions and potential false positives identified through RNA-seq, A-to-I calls were validated by Sanger sequencing. The heights of different base peaks in the Sanger sequence were quantified using ImageJ software (National Institutes of Health, Bethesda, MD, USA).

**3. Treatment with actinomycin D and ribonuclease R**

TE1 or ECA109 cells were exposed to 5 μg/mL actinomycin D or dimethyl sulfoxide (DMSO) for the specified durations, and subsequently, the cells were harvested for RT-qPCR analysis. To selectively degrade linear mRNA, RNase R (Epicentre) was employed.

In brief, RNA was extracted from TE1 or ECA109 cells and divided into two groups. One group underwent digestion with RNase R, while the other group served as a control and was treated with digestion buffer only. The samples were incubated at 37 °C for 15 min, following which the levels of circPDE5A and PDE5A were assessed through RT-qPCR.

**4. RNA-FISH**

The RNA-FISH assay was conducted using a FISH kit (RiboBio, Guangzhou, China) in accordance with the manufacturer's instructions. The subcellular localization of circPDE5A and its level in histopathological sections were determined using a 5'-Cy3-labeled probe designed to target the circPDE5A reverse splice site. Images were captured using a Zeiss LSM 710 laser scanning confocal system (Zeiss, Oberkochen, Germany). The FISH probe sequence for circPDE5A was Cy3-ACCATTTCTCGCTAACGACTG.

**5. Plasmid construction and cell transfection**

The circPDE5A sequence was integrated into the plenti-ciR-copGFP-T2A-puro vector to generate a circPDE5A overexpression vector (IgeBio Technology, Guangzhou, China). Short hairpin RNA (shRNA) targeting the circPDE5A reverse splice site was obtained from IgeBiotech. To validate the presence and function of PDE5A-500aa, three flag-tagged circPDE5A-associated plasmids (circPDE5A-flag, circPDE5A-flag-Mut, and PDE5A-500aa-flag) were constructed (**Table S3**). For investigating the impact of PDE5A-500aa-mediated USP14 on specific ubiquitination types and sites of PIK3IP1, various HA-tagged ubiquitination plasmids (WT, K0, K6, K11, K27, K29, K33, K48, and K63) and lysine site-mutated PIK3IP1 mutants (WT, K93R, K161R, K163R, K164R, K195R, K198R, K201R, K207R, and K234R) were constructed (HanBio Technology, Shanghai, China).

Additionally, plasmids for Myc-tagged PIK3IP1 and flag-tagged USP14 de-ubiquitinating enzyme active site mutation (USP14 C114A) were constructed. The USP14 overexpression plasmid and shRNA targeting USP14 were procured from IgeBiotech. Transfection of cells with these plasmids was carried out using Hieff Trans® Liposomal Transfection Reagent (Yeasen, Shanghai, China) following the manufacturer's instructions. The lentiviral shRNA target sequences are provided in **Table S4**.

**6. Construction of stabilized cell lines**

Lentiviruses were generated by co-transfecting 293T cells with lentiviral vectors (plenti-ciR-circPDE5A-copGFP-T2A-puro, pCDH-CMV-PDE5A-500aa-EF1-copGFP-T2A-puro, pLKO.1-circPED5A-sh1-puro, or pLKO.1-circPED5A-sh2-puro) and lentiviral components (psPAX2 and pMD2.G) at appropriate ratios. The lentivirus-containing supernatants were collected 48 and 72 h post-transfection for infection of ESCC cells. Stable cell lines were established through a 3-day selection period using DMEM complete medium containing 8 μg/mL puromycin. Subsequently, puromycin at a concentration of 4 μg/mL was utilized for the maintenance of the selected cell lines.

**7. Cell proliferation assay**

The proliferation capacity of ESCC cells was assessed using the Cell Counting Kit-8 (CCK8; ApexBio, Houston, TX, USA) following the instructions provided by the reagent manufacturer. Treated ESCC cells were seeded into 96-well plates, and the OD450 absorbance values were recorded at 0, 24, 48, and 72 h of incubation using a microplate reader (Molecular Devices, Shanghai, China).

**8. Plate cloning assay**

To assess plate cloning in ESCC cells, 1,000 treated cells were seeded into six-well plates and cultured for 2 weeks. Afterwards, the cells were fixed using 4% paraformaldehyde for 40 min, followed by staining with crystal violet solution for an additional 40 min. The visible clonal cell colonies were then captured, counted, and subjected to statistical analysis.

**9. 5-Ethynyl-2-deoxyuridine (EdU) Staining**

Cell proliferation status was evaluated using the BeyoClick™ EdU Cell Proliferation Kit with Alexa Fluor 555 (Beyotime, Shanghai, China) in accordance with the manufacturer's instructions. In brief, cells were seeded into 24-well plates and incubated with EdU for 2 h. Subsequently, the cells were fixed with 4% paraformaldehyde for 15 min. Staining was performed using the Click reaction mixture and Hoechst 33342. Finally, the cells were visualized using an Olympus IX73 inverted fluorescence microscope system (Olympus, Tokyo, Japan).

**10. Wound healing assay**

The wound healing assay was employed to evaluate cell motility. ESCC cells were seeded into six-well plates, and once cell confluence reached 95%, cell scratches were created perpendicular to the long axis of the six-well plate using a 200 µL pipette tip. Subsequently, serum-free medium was replaced, and imaging along with migration distance measurements were conducted at 0, 24, and 48 h.

**11. Cell migration and invasion assays**

Cell migration and invasion assays were conducted using Transwell chambers (CORNING, Corning, NY, USA) with an 8 μm pore size, either without or with Matrigel coating on the bottom, to evaluate the migratory and invasive capabilities of cells. In the upper chamber, 200 μL serum-free medium containing 5 × 10^4^ ESCC cells was added, while the lower chamber received 700 μL complete medium containing 10% FBS. Following incubation at 37 °C for 24 h (migration) or 48 h (invasion), cells in the lower chamber were fixed with 4% paraformaldehyde and stained with crystal violet (Beyotime). Photomicrographs of random fields were captured using an orthostatic microscope, and cell counting and statistical analysis were performed using ImageJ software.

**12. Western blot (WB)**

Cell lysis and protein extraction were carried out using RIPA buffer (CWBIO, Beijing, China) supplemented with Protease and Phosphatase Inhibitor Cocktail (New Cell and Molecular Biotech, Suzhou, China). The protein concentration of the samples was quantified using the BCA protein quantification kit (Beyotime). Following separation by sodium dodecyl sulfate-polyacrylamide gel electrophoresis (SDS-PAGE), proteins were transferred to a PVDF membrane. The membranes were initially incubated with a 5% bovine serum albumin solution for 1 h at 25 °C, followed by overnight incubation with primary antibodies at 4 °C. Subsequently, the membrane was incubated with the appropriate secondary antibody for 1 h at 25 °C. Signal detection and image acquisition were accomplished using enhanced chemiluminescence reagent (Vazyme) and the G: BOX gel imaging system (Syngene, Bengaluru, India). The antibodies utilized in this study are detailed in **Table S5**.

**13. IHC**

Immunohistochemical analysis was conducted following standard protocols. In brief, tissues were fixed, dehydrated, embedded, and sectioned using established procedures. Tissue sections were treated with pepsin antigen repair solution (Servicebio, Wuhan, China) at 37 °C for 30 min to restore antigenicity. Subsequently, they were incubated in 3% hydrogen peroxide at 25 °C for 30 min to block endogenous peroxidase. To prevent non-specific binding, the sections were treated with normal goat serum at 37 °C for 30 min and then incubated with primary antibodies at 4 °C overnight. Detection of specifically bound primary antibodies was carried out using HRP-conjugated secondary antibodies and a DAB chromogenic kit (ZSGB-BIO, Beijing, China) according to the manufacturer's instructions. Hematoxylin staining was performed for nucleus visualization. Two independent observers evaluated the degree and intensity of staining for the target proteins, and the integrated optical density of the target proteins was calculated using ImageJ software.

**14. Co-immunoprecipitation (Co-IP) and mass spectrometry**

Immunoprecipitation was conducted using an IP/Co-IP kit (Beyotime) in accordance with the manufacturer's instructions. Briefly, cells were lysed with RIPA lysate, and the supernatants were collected after centrifugation for subsequent IP. The antibody working solution for IP/Co-IP and Protein A+G magnetic beads was incubated for 1 h at 25 °C on an orbital shaker to facilitate antibody binding to the magnetic beads. The protein supernatant was then combined with antibody-adsorbed Protein A+G magnetic beads and subjected to overnight incubation at 4 °C on a rotary mixer. After incubation, the magnetic beads were separated using a magnetic rack, and the supernatant was extracted for SDS-PAGE or WB. Protein bands were visualized by silver staining, and specific bands were manually excised from the gel, followed by digestion with chymotrypsin (Madison, WI, USA). Chromatographic separation was achieved using a nanoliter flow rate Easy nLC 1200 chromatography system (Thermo Scientific). Peptides were separated and analyzed by Data-Dependent Acquisition mass spectrometry using a Q-Exactive Plus mass spectrometer (Thermo Scientific). Database searching and data analysis were performed using Uniprot Protein Data and MaxQuant v2.0.1.0 mass spectrometry database searching software.

**15. Dual-luciferase reporter assay**

The pcDNA3.1(+)-RLuc-MCS-Luc vector (IgeBio Technology) was used to insert the full-length sequence, mutated sequence, and truncated sequence of the predicted IRES of circPDE5A. Subsequently, the constructed reporter vector was transfected into 293T cells. Firefly luciferase and renilla luciferase activities were then measured utilizing the Dual-Glo Fluorophore Lyase Assay Kit (Vazyme).

**16. Immunofluorescence**

Cells were plated in confocal dishes (Corning), fixed with 4% paraformaldehyde for 20 min, and subsequently permeabilized with 0.5% TritonX-100 for 15 min. Following this, cells were blocked with 10% normal anti-goat serum for 30 min at 4 °C, after which they were incubated with the primary antibody overnight at 4 °C. The fluorescent secondary antibody was applied to the cells for 2 h at 25 °C, followed by staining with DAPI solution for 10 min to visualize nuclei. Images were captured using a Zeiss LSM 710 laser scanning confocal system (Zeiss).

**17. Cycloheximide (CHX) chase assay**

To assess the stability of PIK3IP1, the CHX chase assay was employed. Cells overexpressing or with knockdown of circPDE5A were seeded in 6-well plates for 24 h and subsequently treated with CHX (20 μg/mL) for specified durations. Following the CHX treatment, cells from different time points were individually collected, lysed, and subjected to WB to measure the protein levels of PIK3IP1.

**18. In vitro ubiquitination assay**

For the in vitro ubiquitination assay, cells were transiently transfected with plasmids expressing HA-Ub and Myc-PIK3IP1, along with the specified intervening factors. Following treatment with MG132 (MCE, Shanghai, China) for 8 h, total protein was extracted for subsequent IP/Co-IP and WB. Specifically, an anti-Myc antibody and IP/Co-IP kit (Beyotime) were used to isolate PIK3IP1 protein, which was then analyzed by WB using anti-HA or anti-ubiquitin antibody to assess the ubiquitylation status of PIK3IP1.

**19. Flow cytometry**

Six-well plates were plated with 5 × 10^5^ KYSE30 cells. Following a 24-hour incubation period, KYSE30 cells were exposed to PBS, naked circPDE5A-Cy5 plasmid, and NPs loaded with circPDE5A-Cy5 for 4 h. Subsequently, the fluorescence intensity of Cy5 in each group was measured using a BD FACSCANTO™ II flow cytometer.

**Supplementary Tables**

| **Table S1. The top 20 abundant proteins specifically bound to PDE5A-500aa identified by LC-MS/MS analysis.** | | | | |
| --- | --- | --- | --- | --- |
| **IDs** | **Gene names** | **Protein names** | **Mass** | **emPAI** |
| Q96FE7 | PIK3IP1 | Phosphoinositide-3-kinase-interacting protein 1 | 28686 | 2.97 |
| P60174 | TPIS | Triosephosphate isomerase | 26938 | 2.61 |
| P04406 | G3P | Glyceraldehyde-3-phosphate dehydrogenase | 36201 | 2.53 |
| P63261 | ACTG | Actin, cytoplasmic 2 | 42108 | 2.28 |
| P60709 | ACTB | Actin, cytoplasmic 1 | 42052 | 2.12 |
| P00338 | LDHA | L-lactate dehydrogenase A chain | 36950 | 1.71 |
| Q06830 | PRDX1 | Peroxiredoxin-1 | 22324 | 1.63 |
| P07195 | LDHB | L-lactate dehydrogenase B chain | 36900 | 1.28 |
| P62258 | 1433E | 14-3-3 protein epsilon | 29326 | 1.26 |
| P10412 | H14 | Histone H1.4 | 21852 | 1.2 |
| P32119 | PRDX2 | Peroxiredoxin-2 | 22049 | 1.19 |
| P04075 | ALDOA | Fructose-bisphosphate aldolase A | 39851 | 1.15 |
| P62805 | H4 | Histone H4 | 11360 | 1.12 |
| P63104 | 1433Z | 14-3-3 protein zeta/delta | 27899 | 1.01 |
| Q9UQ80 | PA2G4 | Proliferation-associated protein 2G4 | 44101 | 1 |
| Q02539 | H11 | Histone H1.1 | 21829 | 1 |
| Q15717 | ELAV1 | ELAV-like protein 1 | 36240 | 0.93 |
| Q13162 | PRDX4 | Peroxiredoxin-4 | 30749 | 0.89 |
| P00558 | PGK1 | Phosphoglycerate kinase 1 | 44985 | 0.88 |
| P62277 | RS13 | 40S ribosomal protein S13 | 17212 | 0.87 |

| **Table S2. Primer sequences for RT-qPCR** | | |
| --- | --- | --- |
| **Gene name** | **Primer** | **Sequence (5'-3')** |
| GAPDH | Forward | CTCGATGGGTGGAGTCGC |
|  | Reverse | GCATCACCCGGAGGAGAAAT |
| circDDX3Y | Forward | CACATATTGAGAATAAGGGCGCT |
|  | Reverse | TCCCCTTGATCCACTTCCAC |
| circPDE5A | Forward | GAGCTACAGTCGTTAGCGAGA |
|  | Reverse | CCTTCCTTGCACACAGGGAT |
| circPDE5A  (Divergent primers) | Forward | GAGCTACAGTCGTTAGCGAGA |
|  | Reverse | AGGGGCACTGTTATCTGCAC |
| circPDE5A  (Convergent primers) | Forward | CCATCTGCCCAGACCCTTAAA |
|  | Reverse | TGCACAAGGTTGAGGTCAGT |
| linPDE5A | Forward | GCTGCTTGACCTTGCTAGTTT |
|  | Reverse | AAATGGTGCATTTCTGCACTTG |

| **Table S3. The complete sequence of flag-tagged circPDE5A-associated overexpression vectors** | |
| --- | --- |
| **Name** | **Sequence** |
| circPDE5A-flag | AGAAATGGTCAATGCATGGTTTGCGATTACAAGGATGACGACGATAAGTGAGAGAGTTCACACCATCCCTGTGTGCAAGGAAGGTATCAGAGGCCACACCGAATCTTGCTCTTGTCCCTTGCAGCAGAGTCCTCGTGCAGATAACAGTGCCCCTGGAACACCAACCAGGAAAATCTCTGCCTCTGAATTTGACCGGCCTCTTAGACCCATTGTTGTCAAGGATTCTGAGGGAACTGTGAGCTTCCTCTCTGACTCAGAAAAGAAGGAACAGATGCCTCTAACCCCTCCAAGGTTTGATCATGATGAAGGGGACCAGTGCTCAAGACTCTTGGAATTAGTGAAGGATATTTCTAGTCATTTGGATGTCACAGCCTTATGTCACAAAATTTTCTTGCATATCCATGGACTGATATCTGCTGACCGCTATTCCCTGTTCCTTGTCTGTGAAGACAGCTCCAATGACAAGTTTCTTATCAGCCGCCTCTTTGATGTTGCTGAAGGTTCAACACTGGAAGAAGTTTCAAATAACTGTATCCGCTTAGAATGGAACAAAGGCATTGTGGGACATGTGGCAGCGCTTGGTGAGCCCTTGAACATCAAAGATGCATATGAGGATCCTCGGTTCAATGCAGAAGTTGACCAAATTACAGGCTACAAGACACAAAGCATTCTTTGTATGCCAATTAAGAATCATAGGGAAGAGGTTGTTGGTGTAGCCCAGGCCATCAACAAGAAATCAGGAAACGGTGGGACATTTACTGAAAAAGATGAAAAGGACTTTGCTGCTTATTTGGCATTTTGTGGTATTGTTCTTCATAATGCTCAGCTCTATGAGACTTCACTGCTGGAGAACAAGAGAAATCAGGTGCTGCTTGACCTTGCTAGTTTAATTTTTGAAGAACAACAATCATTAGAAGTAATTTTGAAGAAAATAGCTGCCACTATTATCTCTTTCATGCAAGTGCAGAAATGCACCATTTTCATAGTGGATGAAGATTGCTCCGATTCTTTTTCTAGTGTGTTTCACATGGAGTGTGAGGAATTAGAAAAATCATCTGATACATTAACAAGGGAACATGATGCAAACAAAATCAATTACATGTATGCTCAGTATGTCAAAAATACTATGGAACCACTTAATATCCCAGATGTCAGTAAGGATAAAAGATTTCCCTGGACAACTGAAAATACAGGAAATGTAAACCAGCAGTGCATTAGAAGTTTGCTTTGTACACCTATAAAAAATGGAAAGAAGAATAAAGTTATAGGGGTTTGCCAACTTGTTAATAAGATGGAGGAGAATACTGGCAAGGTTAAGCCTTTCAACCGAAATGACGAACAGTTTCTGGAAGCTTTTGTCATCTTTTGTGGCTTGGGGATCCAGAACACGCAGATGTATGAAGCAGTGGAGAGAGCCATGGCCAAGCAAATGGTCACATTGGAGGTTCTGTCGTATCATGCTTCAGCAGCAGAGGAAGAAACAAGAGAGCTACAGTCGTTAGCG |
| circPDE5A-flag-Mut | AGAAACGGTCAATGCATGGTTTGCGATTACAAGGATGACGACGATAAGTGAGAGAGTTCACACCATCCCTGTGTGCAAGGAAGGTATCAGAGGCCACACCGAATCTTGCTCTTGTCCCTTGCAGCAGAGTCCTCGTGCAGATAACAGTGCCCCTGGAACACCAACCAGGAAAATCTCTGCCTCTGAATTTGACCGGCCTCTTAGACCCATTGTTGTCAAGGATTCTGAGGGAACTGTGAGCTTCCTCTCTGACTCAGAAAAGAAGGAACAGATGCCTCTAACCCCTCCAAGGTTTGATCATGATGAAGGGGACCAGTGCTCAAGACTCTTGGAATTAGTGAAGGATATTTCTAGTCATTTGGATGTCACAGCCTTATGTCACAAAATTTTCTTGCATATCCATGGACTGATATCTGCTGACCGCTATTCCCTGTTCCTTGTCTGTGAAGACAGCTCCAATGACAAGTTTCTTATCAGCCGCCTCTTTGATGTTGCTGAAGGTTCAACACTGGAAGAAGTTTCAAATAACTGTATCCGCTTAGAATGGAACAAAGGCATTGTGGGACATGTGGCAGCGCTTGGTGAGCCCTTGAACATCAAAGATGCATATGAGGATCCTCGGTTCAATGCAGAAGTTGACCAAATTACAGGCTACAAGACACAAAGCATTCTTTGTATGCCAATTAAGAATCATAGGGAAGAGGTTGTTGGTGTAGCCCAGGCCATCAACAAGAAATCAGGAAACGGTGGGACATTTACTGAAAAAGATGAAAAGGACTTTGCTGCTTATTTGGCATTTTGTGGTATTGTTCTTCATAATGCTCAGCTCTATGAGACTTCACTGCTGGAGAACAAGAGAAATCAGGTGCTGCTTGACCTTGCTAGTTTAATTTTTGAAGAACAACAATCATTAGAAGTAATTTTGAAGAAAATAGCTGCCACTATTATCTCTTTCATGCAAGTGCAGAAATGCACCATTTTCATAGTGGATGAAGATTGCTCCGATTCTTTTTCTAGTGTGTTTCACATGGAGTGTGAGGAATTAGAAAAATCATCTGATACATTAACAAGGGAACATGATGCAAACAAAATCAATTACATGTATGCTCAGTATGTCAAAAATACTATGGAACCACTTAATATCCCAGATGTCAGTAAGGATAAAAGATTTCCCTGGACAACTGAAAATACAGGAAATGTAAACCAGCAGTGCATTAGAAGTTTGCTTTGTACACCTATAAAAAATGGAAAGAAGAATAAAGTTATAGGGGTTTGCCAACTTGTTAATAAGATGGAGGAGAATACTGGCAAGGTTAAGCCTTTCAACCGAAATGACGAACAGTTTCTGGAAGCTTTTGTCATCTTTTGTGGCTTGGGGATCCAGAACACGCAGATGTATGAAGCAGTGGAGAGAGCCATGGCCAAGCAAATGGTCACATTGGAGGTTCTGTCGTATCATGCTTCAGCAGCAGAGGAAGAAACAAGAGAGCTACAGTCGTTAGCG |
| PDE5A-500aa-flag | GAATTCGCCACCATGGTCAATGCATGGTTTGCTGAGAGAGTTCACACCATCCCTGTGTGCAAGGAAGGTATCAGAGGCCACACCGAATCTTGCTCTTGTCCCTTGCAGCAGAGTCCTCGTGCAGATAACAGTGCCCCTGGAACACCAACCAGGAAAATCTCTGCCTCTGAATTTGACCGGCCTCTTAGACCCATTGTTGTCAAGGATTCTGAGGGAACTGTGAGCTTCCTCTCTGACTCAGAAAAGAAGGAACAGATGCCTCTAACCCCTCCAAGGTTTGATCATGATGAAGGGGACCAGTGCTCAAGACTCTTGGAATTAGTGAAGGATATTTCTAGTCATTTGGATGTCACAGCCTTATGTCACAAAATTTTCTTGCATATCCATGGACTGATATCTGCTGACCGCTATTCCCTGTTCCTTGTCTGTGAAGACAGCTCCAATGACAAGTTTCTTATCAGCCGCCTCTTTGATGTTGCTGAAGGTTCAACACTGGAAGAAGTTTCAAATAACTGTATCCGCTTAGAATGGAACAAAGGCATTGTGGGACATGTGGCAGCGCTTGGTGAGCCCTTGAACATCAAAGATGCATATGAGGATCCTCGGTTCAATGCAGAAGTTGACCAAATTACAGGCTACAAGACACAAAGCATTCTTTGTATGCCAATTAAGAATCATAGGGAAGAGGTTGTTGGTGTAGCCCAGGCCATCAACAAGAAATCAGGAAACGGTGGGACATTTACTGAAAAAGATGAAAAGGACTTTGCTGCTTATTTGGCATTTTGTGGTATTGTTCTTCATAATGCTCAGCTCTATGAGACTTCACTGCTGGAGAACAAGAGAAATCAGGTGCTGCTTGACCTTGCTAGTTTAATTTTTGAAGAACAACAATCATTAGAAGTAATTTTGAAGAAAATAGCTGCCACTATTATCTCTTTCATGCAAGTGCAGAAATGCACCATTTTCATAGTGGATGAAGATTGCTCCGATTCTTTTTCTAGTGTGTTTCACATGGAGTGTGAGGAATTAGAAAAATCATCTGATACATTAACAAGGGAACATGATGCAAACAAAATCAATTACATGTATGCTCAGTATGTCAAAAATACTATGGAACCACTTAATATCCCAGATGTCAGTAAGGATAAAAGATTTCCCTGGACAACTGAAAATACAGGAAATGTAAACCAGCAGTGCATTAGAAGTTTGCTTTGTACACCTATAAAAAATGGAAAGAAGAATAAAGTTATAGGGGTTTGCCAACTTGTTAATAAGATGGAGGAGAATACTGGCAAGGTTAAGCCTTTCAACCGAAATGACGAACAGTTTCTGGAAGCTTTTGTCATCTTTTGTGGCTTGGGGATCCAGAACACGCAGATGTATGAAGCAGTGGAGAGAGCCATGGCCAAGCAAATGGTCACATTGGAGGTTCTGTCGTATCATGCTTCAGCAGCAGAGGAAGAAACAAGAGAGCTACAGTCGTTAGCGAGAAATGGTCAATGCATGGTTTGCGATTACAAGGATGACGACGATAAGTGAGGATCC |

| **Table S4. The sequence of shRNAs** | |
| --- | --- |
| **Name** | **Sequence (5'-3')** |
| circPDE5A-shRNA1-Sense | GTCGTTAGCGAGAAATGGTCA |
| circPDE5A-shRNA1-antiSense | TGACCATTTCTCGCTAACGAC |
| circPDE5A-shRNA2-Sense | CTACAGTCGTTAGCGAGAAAT |
| circPDE5A-shRNA2-antiSense | ATTTCTCGCTAACGACTGTAG |
| circPDE5A-shRNA3-Sense | GCTACAGTCGTTAGCGAGAAA |
| circPDE5A-shRNA3-antiSense | TTTCTCGCTAACGACTGTAGC |
| PIK3IP1-shRNA-Sense | CATTACCATGATGGTGATCAT |
| PIK3IP1-shRNA- antiSense | ATGATCACCATCATGGTAATG |
| USP14-shRNA-Sense | CCCAAGATTCAGCAGTCAGAT |
| USP14-shRNA- antiSense | ATCTGACTGCTGAATCTTGGG |

| **Table S5. Antibodies used in this study** | | |
| --- | --- | --- |
| **Antibody** | **Manufacturers** | **Applications** |
| E-Cadherin | #3195, Cell Signaling Technology, Beverly, MA, USA | 1:1000 for WB  1:400 for IHC |
| Claudin-1 | #13255, Cell Signaling Technology, Beverly, MA, USA | 1:1000 for WB |
| N-Cadherin | #13116, Cell Signaling Technology, Beverly, MA, USA | 1:1000 for WB  1:100 for IHC |
| Vimentin | #5741, Cell Signaling Technology, Beverly, MA, USA | 1:1000 for WB |
| Snail | #3879, Cell Signaling Technology, Beverly, MA, USA | 1:1000 for WB |
| Slug | #9585, Cell Signaling Technology, Beverly, MA, USA | 1:1000 for WB |
| PDE5A | Cat No. 22624-1-AP, Proteintech, Chicago, USA | 1:1000 for WB |
| Flag-Tag | #14793, Cell Signaling Technology, Beverly, MA, USA | 1:50 for IP  1:1000 for WB  1:400 for IF |
| PIK3IP1 | #DF12453, Affinity, USA | 1:1000 for WB  1:100 for IHC  1:100 for IF |
| PIK3IP1 | Cat No. 16826-1-AP, Proteintech, Chicago, USA | 1:50 for IP |
| P110α | YT3709, Immunoway, CA, USA | 1:1000 for WB  1:100 for IHC |
| AKT | YT0185, Immunoway, CA, USA | 1:1000 for WB |
| p-AKT | YP0006, Immunoway, CA, USA | 1:1000 for WB  1:100 for IHC |
| mTOR | YT2913, Immunoway, CA, USA | 1:1000 for WB |
| p-mTOR | YP0176, Immunoway, CA, USA | 1:1000 for WB  1:100 for IHC |
| c-Myc | ab32072, Abcam, Cambridge, UK | 1:1000 for WB |
| MMP2 | #4022, Cell Signaling Technology, Beverly, MA, USA | 1:1000 for WB |
| MMP9 | #13667, Cell Signaling Technology, Beverly, MA, USA | 1:1000 for WB |
| GAPDH | #2118, Cell Signaling Technology, Beverly, MA, USA | 1:1000 for WB |
| HA-Tag | #3724, Cell Signaling Technology, Beverly, MA, USA | 1:1000 for WB |
| Myc-Tag | #2278, Cell Signaling Technology, Beverly, MA, USA | 1:1000 for WB |
| USP14 | ab192618, Abcam, Cambridge, UK | 1:1000 for WB |
| Ki-67 | ET1609-34, HUABIO, Hangzhou, China | 1:100 for IHC |
| HRP-linked anti-rabbit IgG | #RS0002, Immunoway, CA, USA | 1:10000 for WB |
| HRP-linked anti-mouse IgG | #RS0001, Immunoway, CA, USA | 1:10000 for WB |
| Anti-mouse IgG (H+L) (Alexa Fluor® 594 Conjugate) | #ZF-0513, ZSGB-BIO, Beijing, China | 1:100 for IF |
| Anti-rabbit IgG (H+L) (Alexa Fluor® 488 Conjugate) | #ZF-0513, ZSGB-BIO, Beijing, China | 1:100 for IF |

**Supplementary figures
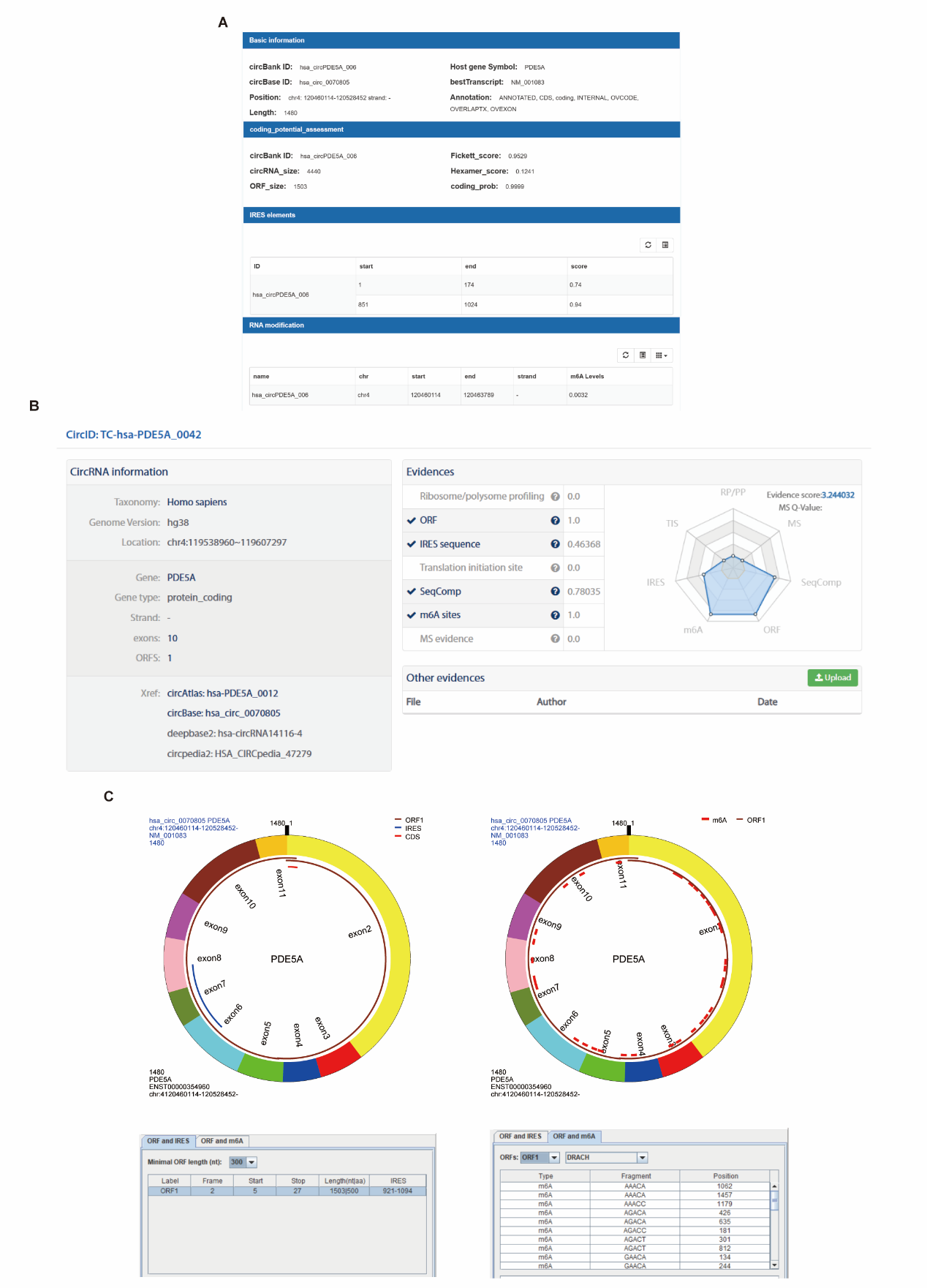
**

**Figure S1. CircPDE5A contains critical elements encoding proteins.**

(**A–C**) Prediction of open reading frames (ORFs), N6-adenylate methylation (m6A), and internal ribosomal entry sites (IRES) of circPDE5A using circBank (**A**), TransCirc (**B**), and circPrimer (**C**) databases.


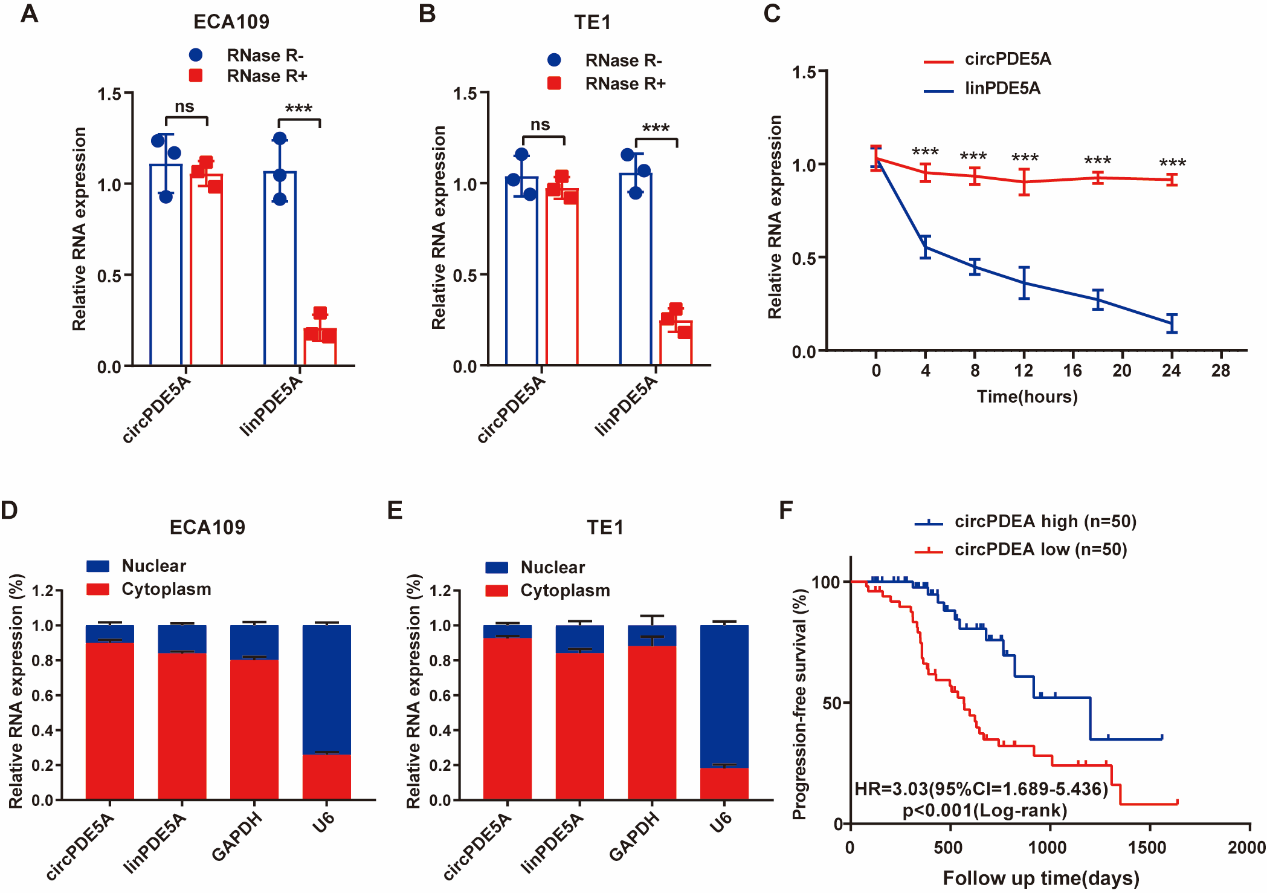
**Figure S2. Stability and prognostic value of circPDE5A.**

(**A–B**) tRNA from ECA109 (**A**) or TE1 (**B**) cells treated with or without RNase R, followed by analysis of circPDE5A and PDE5A linear mRNA level using RT-qPCR. (**C**) RT-qPCR analysis of circPDE5A and PDE5A linear mRNA level in actinomycin D-treated ESCC cells at the indicated time points. (**D–E**) RT-qPCR analysis of circPDE5A localization in the cytoplasm or nucleus of ECA109 (**D**) or TE1 (**E**) cells. GADPH served as a cytoplasmic localization marker, and U6 served as a nuclear localization marker. (**F**) Kaplan-Meier analysis of the correlation between circPDE5A level and progression-free survival time (PFS) in patients with ESCC. ****P* < 0.005. ns, not significant.


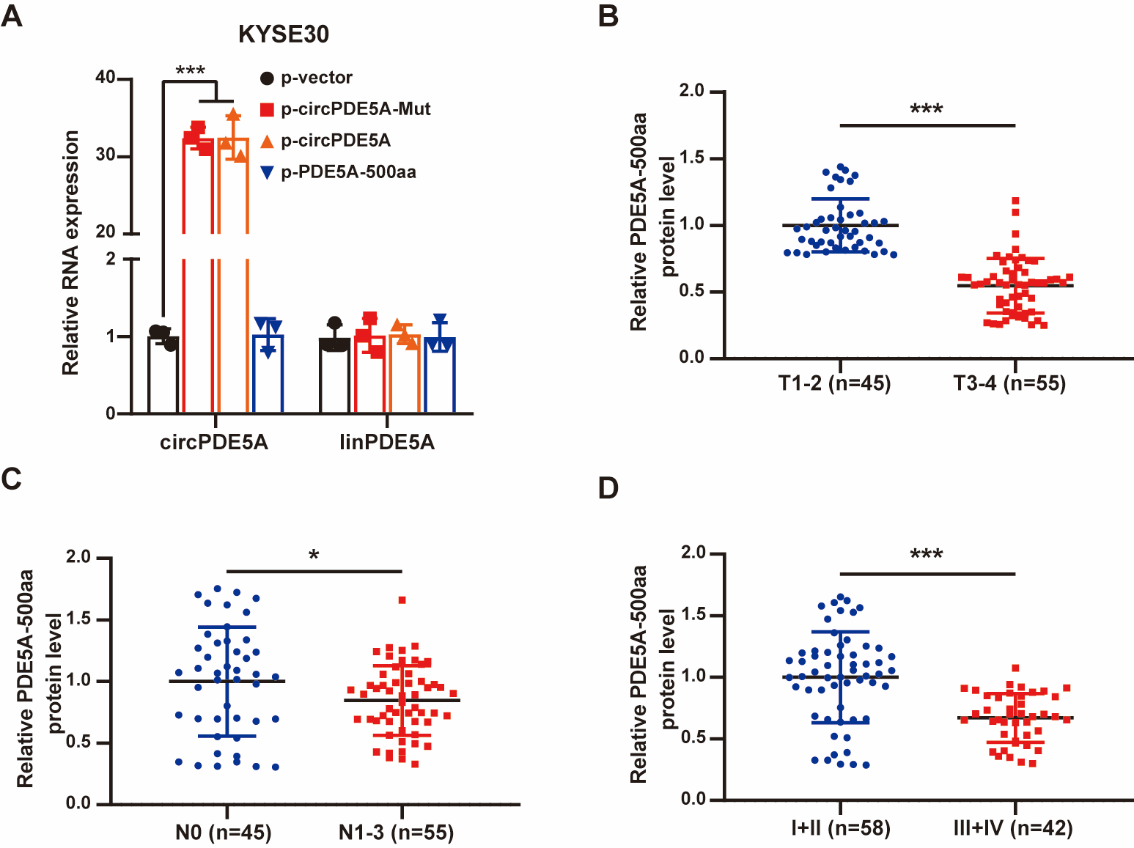


**Figure S3. Clinical significance of PDE5A-500aa.**

(**A**) RT-qPCR detection of linear PDE5A or circPDE5A level in KYSE30 cells treated with p-vector, p-circPDE5A-Mut, p-circPDE5A, or p-PDE5A-500aa, respectively. (**B–D**) Relative levels of PDE5A-500aa protein in ESCC tissues with different T (**B**), N (**C**), and TNM (**D**) staging were detected by WB. **P* < 0.05. ****P* < 0.005.


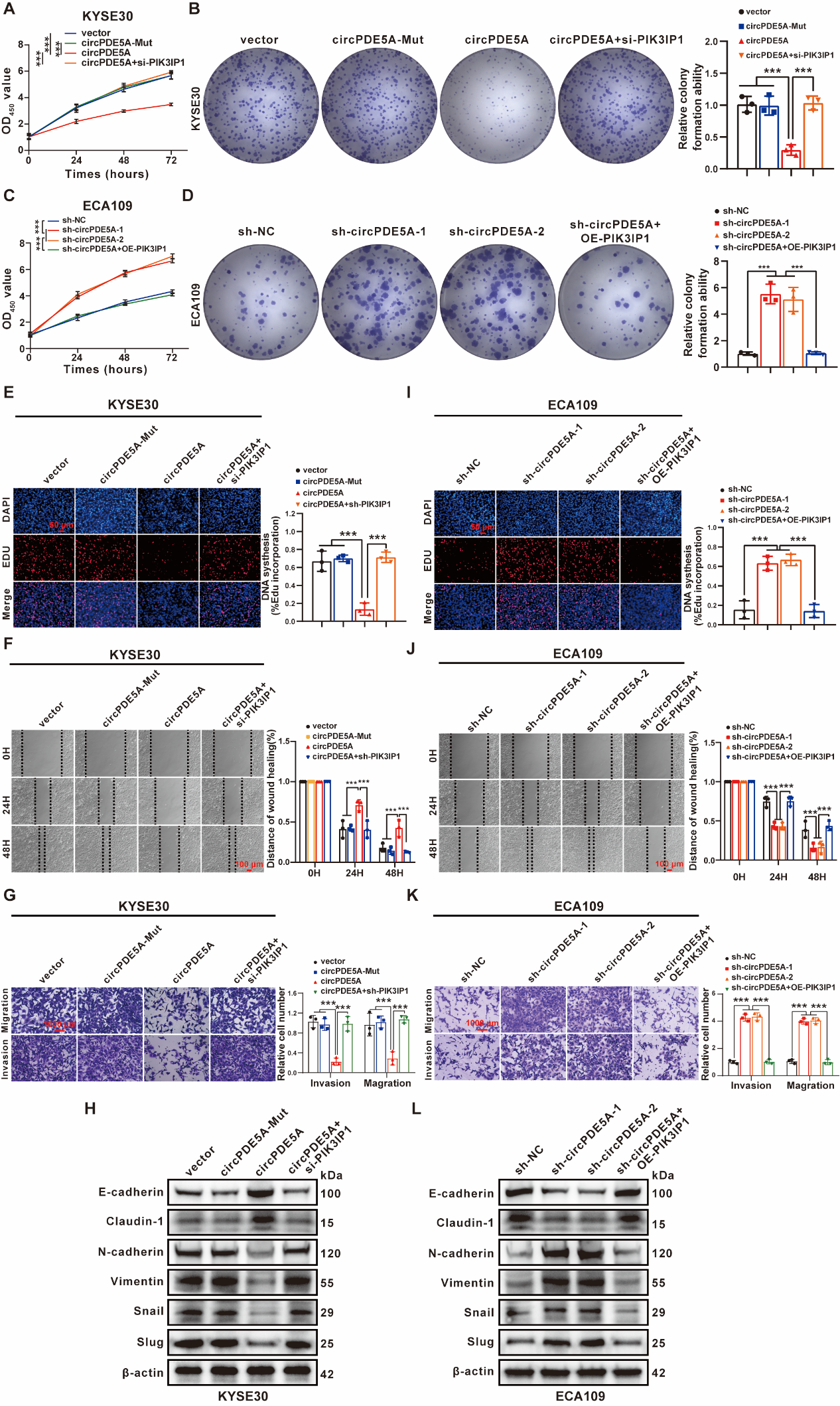


**Figure S4. PDE5A-500aa inhibits ESCC cell proliferation and metastasis by regulating PIK3IP1.**

(**A–B, E**) The proliferation ability of KYSE30 cells assessed by CCK8 (**A**), plate cloning (**B**), and EdU (**E**) assays after treatment with vector, circPDE5A-Mut, circPDE5A, and circPDE5A+sh-PIK3IP1 plasmids. Bar in (**E**) represents 50 μm. (**C–D, I**) The proliferation ability of ECA109 cells assessed by CCK8 (**C**), plate cloning (**D**), and EdU (**I**) assays after treatment with sh-NC, sh-circPDE5A-1, sh-circPDE5A-2, and sh-circPDE5A+OE-PIK3IP1 plasmids. Bar in (**I**) represents 50 μm. (**F**) Motility of KYSE30 cells assessed by wound healing assay after treatment with vector, circPDE5A-Mut, circPDE5A, and circPDE5A+sh-PIK3IP1 plasmids. Bar represents 100 μm. (**G**) Migration and invasion ability of KYSE30 cells assessed by transwell assay after treatment with vector, circPDE5A-Mut, circPDE5A, and circPDE5A+sh-PIK3IP1 plasmids. Bar represents 1,000 μm. (**H**) EMT marker protein levels in KYSE30 cells detected by WB after treatment with vector, circPDE5A-Mut, circPDE5A, and circPDE5A+sh-PIK3IP1 plasmids. (**J**) Motility of ECA109 cells assessed by wound healing assay after treatment with sh-NC, sh-circPDE5A-1, sh-circPDE5A-2, and sh-circPDE5A+OE-PIK3IP1 plasmids. Bar represents 100 μm. (**K**) Migration and invasion ability of ECA109 cells assessed by transwell assay after treatment with sh-NC, sh-circPDE5A-1, sh-circPDE5A-2, and sh-circPDE5A+OE-PIK3IP1 plasmids. Bar represents 1,000 μm. (**L**) EMT marker protein levels in ECA109 cells detected by WB after treatment with sh-NC, sh-circPDE5A-1, sh-circPDE5A-2, and sh-circPDE5A+OE-PIK3IP1 plasmids. ****P* < 0.005.


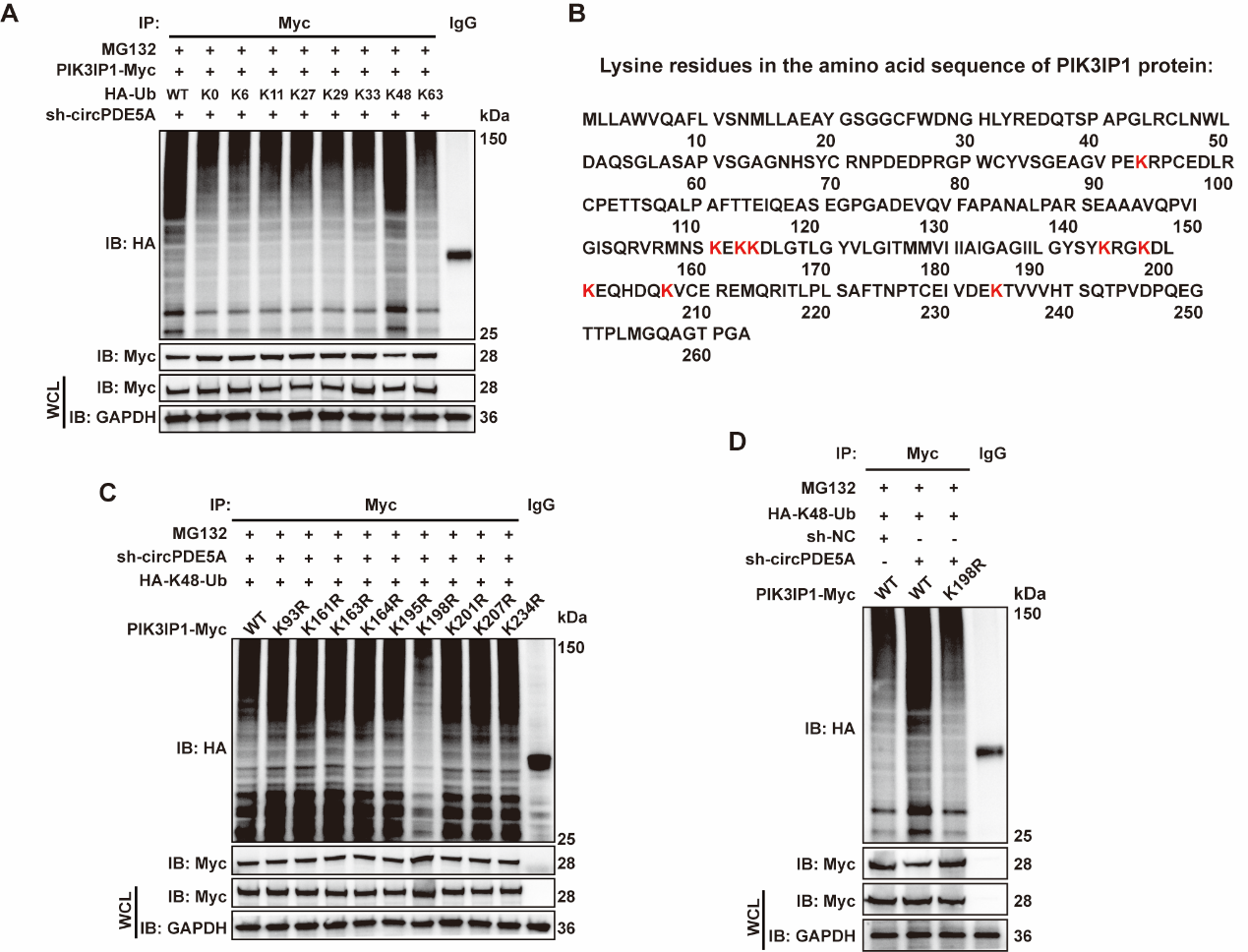


**Figure S5. PDE5A-500aa promotes the de-ubiquitinating activity of PIK3IP1.**

(**A**) IP and WB detection of the ubiquitination level of the PIK3IP1 protein in circPDE5A knockdown ESCC cells co-transfected with HA-tagged different ubiquitin chain vectors (WT, K0, K6, K11, K27, K29, K33, K48, and K63) with Myc-PIK3IP1 and treated with MG132. (**B**) Amino acid sequence and lysine site of the PIK3IP1 protein. (**C**) ESCC cells are co-transfected with HA-K48-Ub and sh-circPDE5A and treated with MG132. The ubiquitination level of PIK3IP1 protein detected by IP and WB after treatment with different Myc-tagged PIK3IP1 lysine site-indicated mutants, respectively. (**D**) ESCC cells are transfected with HA-K48-Ub and treated with MG132. The ubiquitination level of PIK3IP1 protein detected by IP and WB after treatment with sh-NC, sh-circPDE5A, and sh-circPDE5A+Myc-PIK3IP1 (K198R) plasmids, respectively.


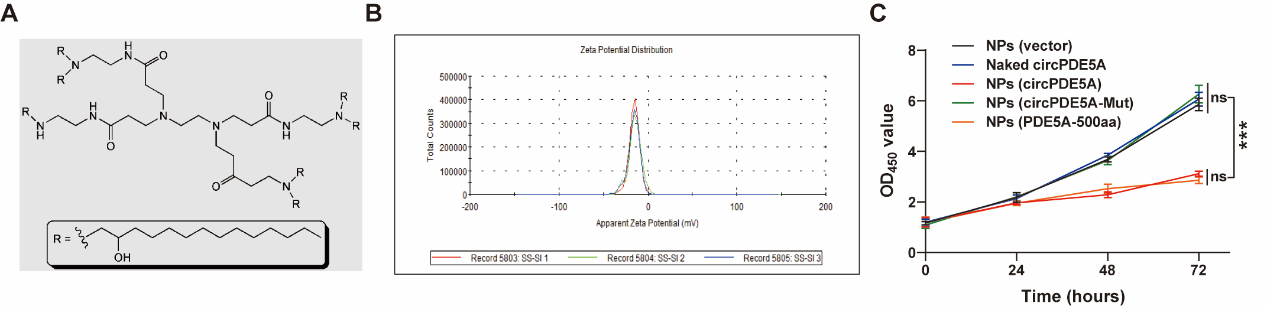


**Figure S6. NP-mediated upregulation of circPDE5A and PDE5A-500aa inhibits ESCC cell proliferation in vitro.**

(**A**) Chemical structure of amphiphilic cationic lipid G0-C14. (**B**) Zeta potential profile of the NPs (circPDE5A). (**C**) Proliferative capacity of ESCC cells assessed by CCK8 assay after treatment with NPs (vector), naked circPDE5A plasmid, NPs (circPDE5A), NPs (circPDE5A-Mut), or NPs (PDE5A-500aa), respectively. ****P* < 0.005. ns, not significant.


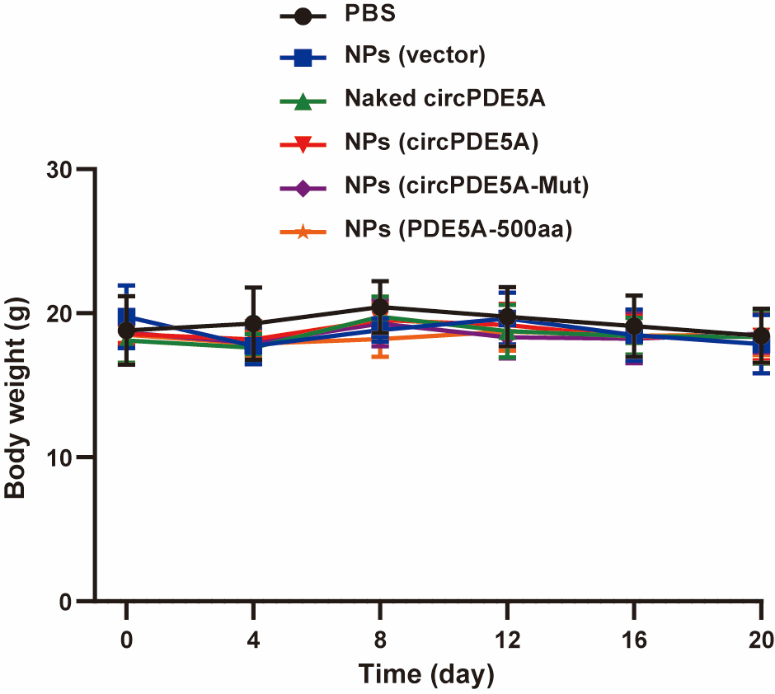


**Figure S7.** Body weights of KYSE30 subcutaneous xenograft tumor mice measured after treatment with NPs (vector), naked circPDE5A plasmid, NPs (circPDE5A), NPs (circPDE5A-Mut), or NPs (PDE5A-500aa), respectively.


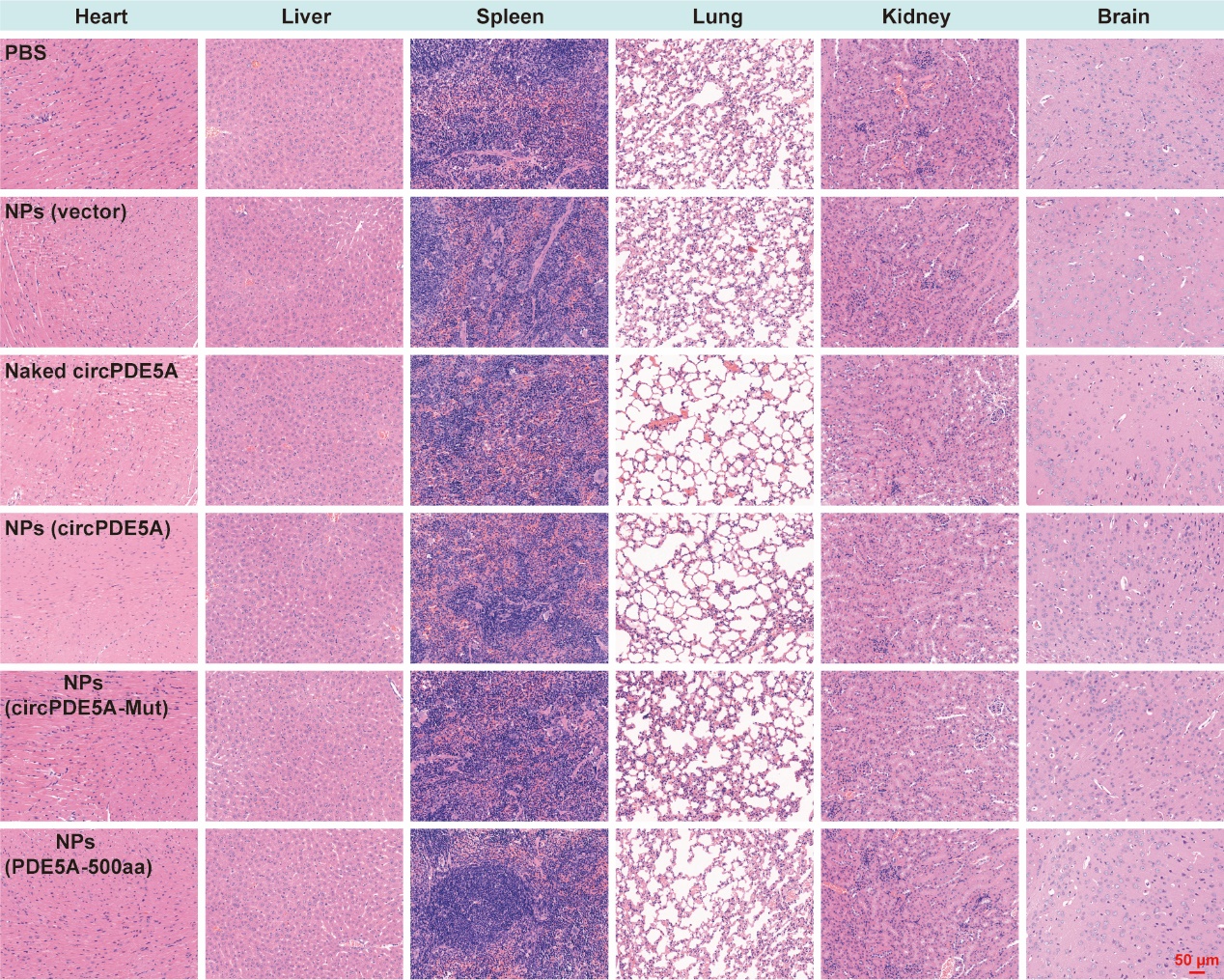


**Figure S8.** HE staining of major organs of KYSE30 subcutaneous xenograft tumor mice after treatment with NPs (vector), naked circPDE5A plasmid, NPs (circPDE5A), NPs (circPDE5A-Mut), or NPs (PDE5A-500aa), respectively. Bar represents 50 μm.


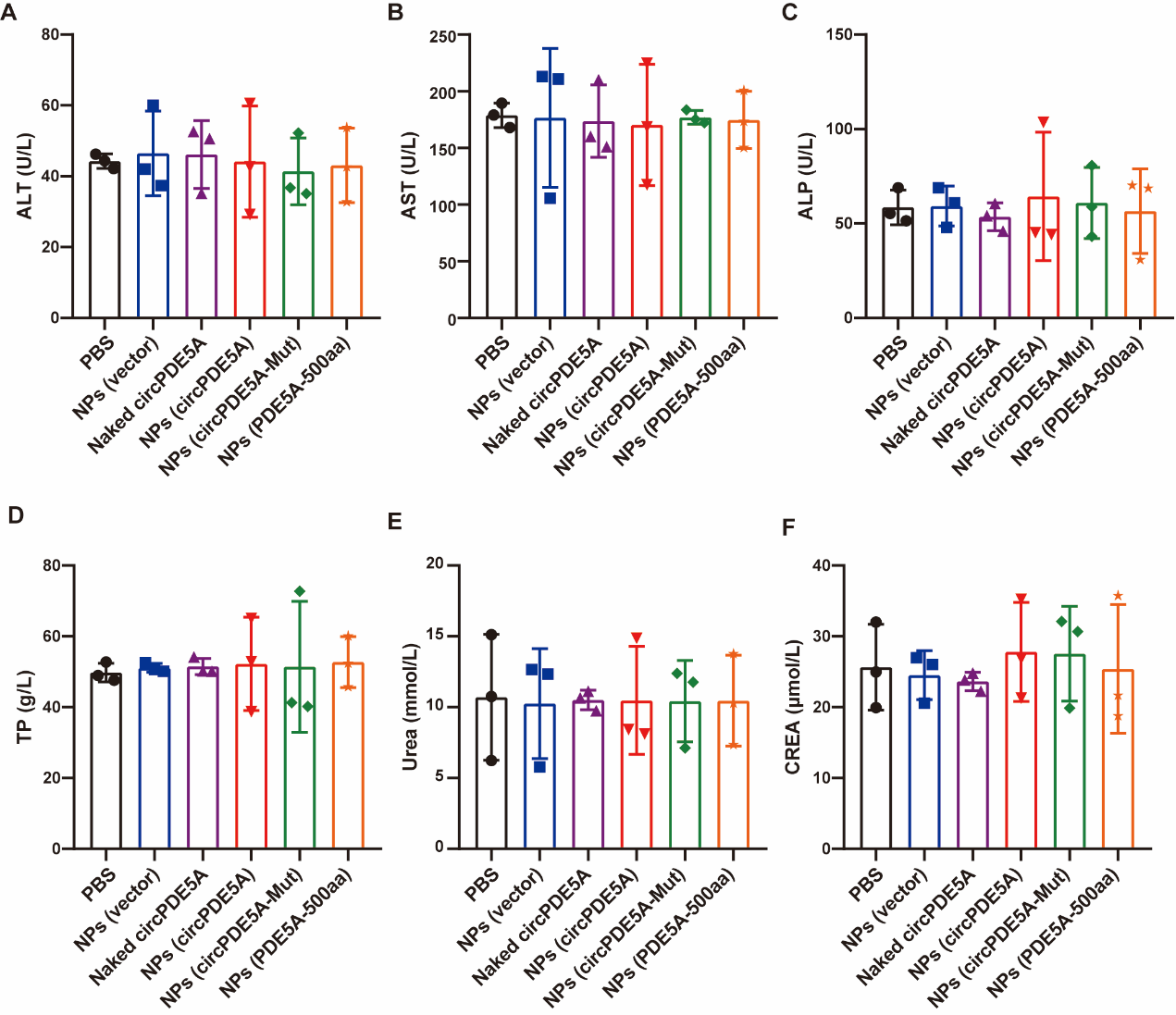


**Figure S9.** Serum alanine aminotransferase (ALT), aspartate aminotransferase (AST), alkaline phosphatase (ALP), total protein (TP), urea, and creatinine (CREA) levels of KYSE30 subcutaneous xenograft tumor-bearing mice determined after treatment of the mice with NPs (vector), naked circPDE5A plasmid, NPs (circPDE5A), NPs (circPDE5A-Mut), or NPs (PDE5A-500aa), respectively.


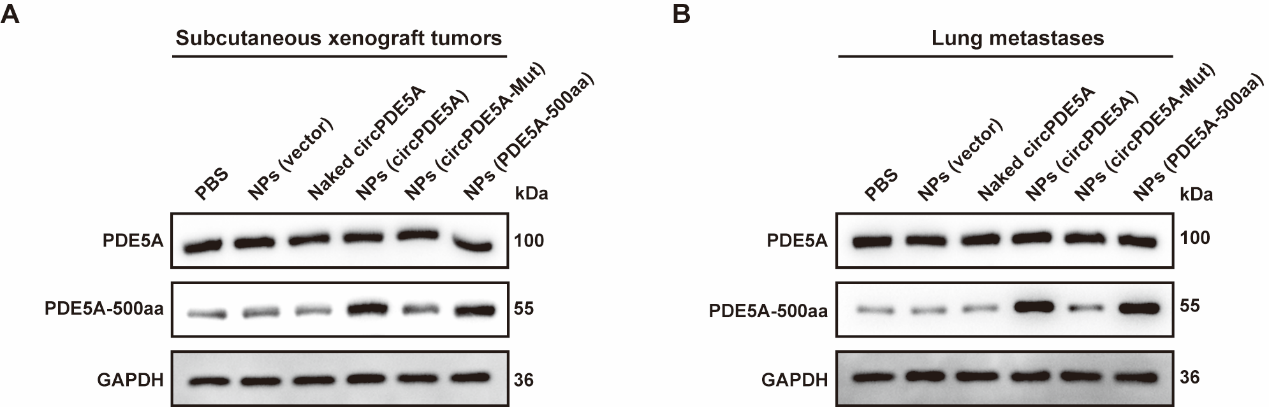


**Figure S10.** (**A**) Detection of PDE5A and PDE5A-500aa expression in subcutaneous xenograft tumors using WB. (**B**) Detection of PDE5A and PDE5A-500aa expression in lung metastases using WB.
